# Generation of a conditional Flpo/FRT mouse model expressing constitutively active TGFβ in fibroblasts

**DOI:** 10.1038/s41598-020-60272-3

**Published:** 2020-03-03

**Authors:** Victoire Cardot-Ruffino, Véronique Chauvet, Cassandre Caligaris, Adrien Bertrand-Chapel, Nicolas Chuvin, Roxane M. Pommier, Ulrich Valcourt, David F. Vincent, Sylvie Martel, Sophie Aires, Bastien Kaniewski, Pierre Dubus, Philippe Cassier, Stéphanie Sentis, Laurent Bartholin

**Affiliations:** 1 INSERM U1052, Centre de Recherche en Cancérologie de Lyon, F-69000 Lyon, France; 2 CNRS UMR5286, Centre de Recherche en Cancérologie de Lyon, F-69000 Lyon, France; 3 Université de Lyon, F-69000 Lyon, France; 4 Université Lyon 1, F-69000 Lyon, France; 5 Centre Léon Bérard, F-69008 Lyon, France; 6 Present Address: Clinical Research Division, Fred Hutchinson Cancer Research Center, Seattle, WA USA; 7 Present Address: Laboratoire de Biologie Tissulaire et Ingénierie Thérapeutique, UMR 5305, CNRS - Université Lyon 1, Institut de Biologie et Chimie des Protéines, SFR BioSciences Gerland-Lyon Sud, 7 passage du Vercors, F-69367 Lyon, France; 8 Present Address: Beatson Institute for Cancer Research, Switchback Road, G61 1BD Glasgow, Scotland; 9 INSERM, Univ Bordeaux UMR1053 Bordeaux Research in Translational Oncology, F-33000 Bordeaux, France; 10 CHU de Bordeaux, F-33000 Bordeaux, France; 11 Departement d’Oncologie Médicale, Centre Léon Bérard, Lyon, 69008 France

**Keywords:** Animal breeding, Genomic engineering

## Abstract

Transforming growth factor (TGFβ) is a secreted factor, which accumulates in tissues during many physio- and pathological processes such as embryonic development, wound healing, fibrosis and cancer. In order to analyze the effects of increased microenvironmental TGFβ concentration *in vivo*, we developed a conditional transgenic mouse model (Flpo/Frt system) expressing bioactive TGFβ in fibroblasts, a cell population present in the microenvironment of almost all tissues. To achieve this, we created the genetically-engineered [Fsp1-Flpo; ^FSF^TGFβ^CA^] mouse model. The *Fsp1-Flpo* allele consists in the Flpo recombinase under the control of the *Fsp1* (fibroblast-specific promoter 1) promoter. The ^*FSF*^*TGFβ*^*CA*^ allele consists in a transgene encoding a constitutively active mutant form of TGFβ (*TGFβ*^*CA*^) under the control of a Frt-STOP-Frt (FSF) cassette. The ^*FSF*^*TGFβ*^*CA*^ allele was created to generate this model, and functionally validated by *in vitro*, *ex vivo* and *in vivo* techniques. [Fsp1-Flpo; ^FSF^TGFβ^CA^] animals do not present any obvious phenotype despite the correct expression of *TGFβ*^*CA*^ transgene in fibroblasts. This [Fsp1-Flpo; ^FSF^TGFβ^CA^] model is highly pertinent for future studies on the effect of increased microenvironmental bioactive TGFβ concentrations in mice bearing Cre-dependent genetic alterations in other compartments (epithelial or immune compartments for instance). These dual recombinase system (DRS) approaches will enable scientists to study uncoupled spatiotemporal regulation of different genetic alterations within the same mouse, thus better replicating the complexity of human diseases.

## Introduction

TGFβ (transforming growth factor beta) is the archetype of the transforming growth factor superfamily^1^. TGFβ (3 isoforms, TGFβ1-2-3) is secreted as a latent complex in which the mature entity remains non-covalently bound to its pro-domain called Latency-Associated Peptide (LAP). During physiological (*e.g*. embryonic development, wound healing, immunosuppression) and pathological processes (*e.g*. fibrosis, cancer), bioactive TGFβ is released from the latent complex to further modulate the activity or the differentiation of surrounding cells. The molecular processes regulating this activation are scarcely understood, the greatest proportion of secreted TGFβ being inactive^2^. After activation, TGFβ signaling occurs through a heterotetrameric receptor complex composed of two subunits, the type I and type II TGFβ receptors (TβRI and TβRII, respectively). Upon binding to its receptors, TGFβ enables TβRII to transphosphorylate TβRI, which in turn activates the canonical Smad pathway (by phosphorylating SMAD2 and SMAD3 transcription factors that further interact with SMAD4 to accumulate inside the nucleus) and other signaling pathways (MAPK, RhoA, and Pi3K/Akt)^3,4^. The effects of a constitutive inhibition of TGFβ signaling has been studied by many approaches using genetically-engineered mice with genes knocked out, deleted, mutated, or dominant-negative mutated. These alterations result in severe phenotypes during development or after birth usually leading to death as observed in TGFβ-KO, TβR-KO and Smad-KO mice^5^. Organ-specific inactivation of TGFβ signaling using the Cre/lox system circumvents these developmental defects, unveiling its crucial role in cell homeostasis and pathogenesis. For instance, during tumorigenesis, loss-of-function of TGFβ signaling results mostly in increased proliferation and tumorigenesis^5^. Conversely, TGFβ gain-of-function has scarcely been explored. Organ-specific activation of TGFβ signaling (constitutively active ligand overexpression or expression of a constitutively active receptor) leads to developmental defects in the mammary gland and skin^6,7^ and to fibrosis in the liver and lungs^8–10^. Few years ago, we created the first conditional constitutively active TβRI receptor allele in mice (^*LSL*^*TβRI*^*CA*^ allele)^11,12^). While constitutive activation of this transgene is embryonically lethal^13^, we showed that it could potentiate *KRAS*^*G12D*^-driven pancreatic transformation when targeted in pancreatic epithelial cell lineages^13^. Targeted expression of *TβRI*^*CA*^ in other organs such as the ovaries^14,15^, uterus^16^, T cells^17^, testis^18^ and liver^19^ results in a large panel of defects in homeostasis and differentiation. Considering the crucial role of TGFβ as a secreted factor in the microenvironment, we created in the present study an original mouse model using the Flp/Frt recombination system^20^ to conditionally express constitutively active TGFβ (^*FSF*^*TGFβ*^*CA*^) in the fibroblastic compartment (*Fsp1-Flpo*), a cell population present in the microenvironment of almost all tissues. We chose the Flp/Frt approach with the final goal of combining this system with the Cre/lox system in dual recombination system (DRS models) in order to explore the effect of increased microenvironmental bioactive TGFβ as observed in many physiopathological contexts.

## Results

**Figure 1:**
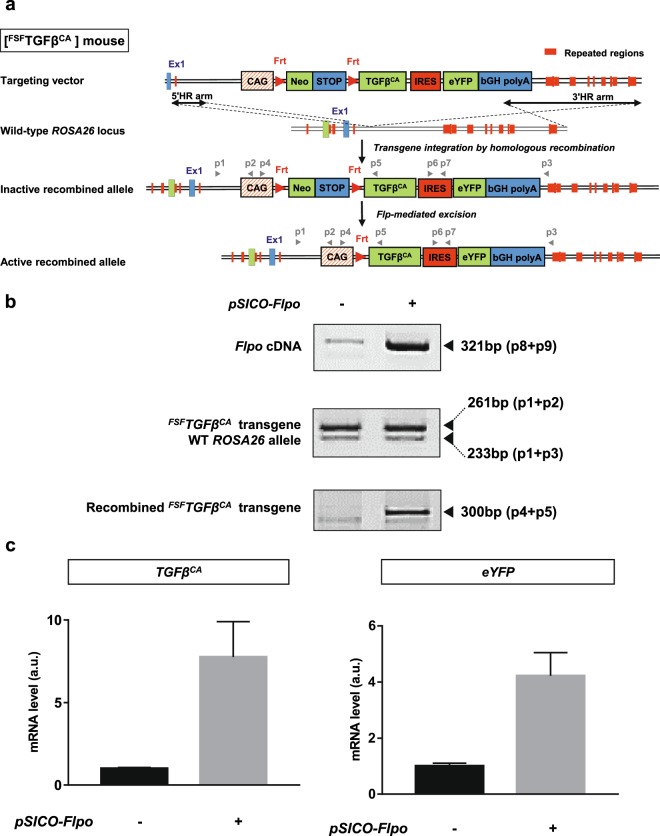
Generation of the [^FSF^TGFβ^CA^] mouse strain. (**a**) Transgenesis strategy to generate the [^FSF^TGFβ^CA^] mouse strain. Site-directed *TGFβ*^*CA*^ transgene integration by homologous recombination into the *ROSA26* locus and Flp-mediated excision of the transcriptional “Stop” allowing *TGFβ*^*CA*^ transgene expression are represented. Primers (p) used for PCR (panel b) and RT-PCR (panel c) are represented by grey arrowheads and detailed in Table 1. CAG, composite constitutive human cytomegalovirus enhancer and chicken beta-actin promoter; Neo, neomycin antibiotic resistance cassette; IRES, internal ribosome entry site; eYFP, enhanced yellow fluorescent protein; bGH polyA, bovine growth hormone polyadenylation signal. (**b**) PCR on genomic DNA prepared from [^FSF^TGFβ^CA^] murine primary ear skin fibroblasts transfected either with the pSICO-Flpo plasmid or an empty plasmid to detect the Flpo sequence, the unrecombined ^*FSF*^*TGFβ*^*CA*^, and the recombined ^*FSF*^*TGFβ*^*CA*^ alleles. (**c**) Quantitative RT-PCR on total mRNA prepared from [^FSF^TGFβ^CA^] murine primary ear skin fibroblasts transfected either with the pSICO-Flpo plasmid or an empty plasmid to detect *TGFβ*^*CA*^ (left panel) and *eYFP* (right panel) mRNA. In b and c, one representative experiment performed 3 times with fibroblasts from different mice is presented. In c, Prism 7.0 (Graphpad) was used to create graphs and the error bars represent the standard deviation from technical duplicates.

We engineered the [^FSF^TGFβ^CA^] mouse strain by homologous recombination in ES cells at the *ROSA26* locus. Details of the experimental procedure to generate this strain are available in the experimental methods section (see Supplementary Figs. S1,2,3,4). Briefly, we constructed a recombinant targeting vector expressing the ^*FSF*^*TGFβ*^*CA*^ transgene and encoding a Flp-conditional (FSF, Frt-STOP-Frt cassette) constitutively active (CA) *TGFβ1* mutant (*TGFβ*^*CA*^) (Fig. 1a). *TGFβ*^*CA*^ encodes a modified secreted polypeptide in which two cysteine residues (Cys223 and Cys225) are substituted with serine residues^21^, preventing the formation of the inactive LAP-TGFβ complex, thus driving the direct secretion of a bioactive TGFβ. In the presence of a recombinase of the Flp family, the transcriptional STOP signal can be excised and the *TGFβ*^*CA*^ transgene expressed under the control of the ubiquitous cytomegalovirus (CMV) early enhancer/chicken β-actin promoter (CAG). To facilitate the detection of cells expressing the transgene, a DNA sequence coding a fluorescent protein (eYFP) under the control of an internal ribosome entry site (IRES) was fused to the 3′ end of *TGFβ*^*CA*^ cDNA. In order to validate the conditional expression of the *TGFβ*^*CA*^ transgene *ex vivo*, we prepared ear skin primary fibroblasts from [^FSF^TGFβ^CA^] mice. These cells were transiently transfected with an empty plasmid or a pSICO-Flpo plasmid expressing the Flpo recombinase under the control of the CMV promoter. We observed the recombined transgene (excision of the STOP cassette) by PCR on genomic DNA exclusively when the Flpo recombinase was expressed (Fig. 1b). A RT-PCR was performed on total RNA prepared from these transfected cells and revealed an increase in *TGFβ*^*CA*^ (Fig. 1c, left panel) and *eYFP* (Fig. 1c, right panel) mRNA levels in cells transfected with the pSICO-Flpo plasmid.

**Figure 2:**
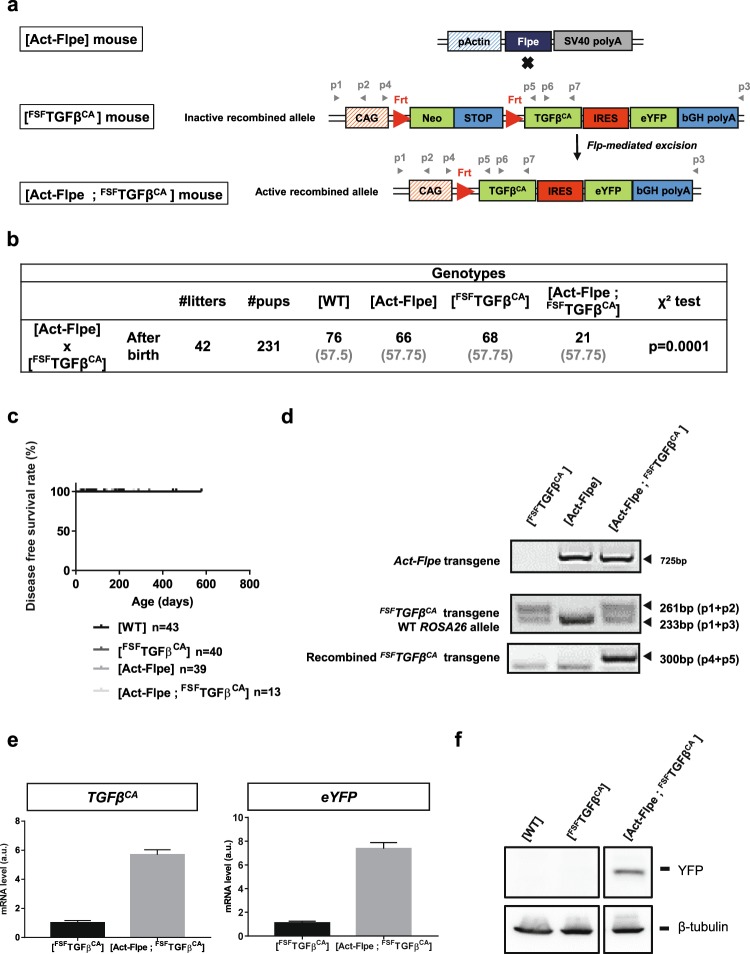
*In vivo* functional validation of the ^*FSF*^*TGFβ*^*CA*^ allele. (**a**) Breeding strategy ([Act-Flpe] x [^FSF^TGFβ^CA^]) to generate [Act-Flpe; ^FSF^TGFβ^CA^] individuals. Primers (p) used for DNA genotyping (panel d) and RT-PCR (panel e) are represented by grey arrowheads. (**b**) [^FSF^TGFβ^CA^] were crossed with [Act-Flpe] mice. Total numbers (black) and expected numbers (grey) of litters, pups, and offspring genotype distribution are presented. A χ² test was performed to statistically confirm the “loss” of a significant proportion of [Act-Flpe; ^FSF^TGFβ^CA^] individuals after birth. The Chi2 were calculated using Excel workbook developed by Montoliu^36^. (**c**) Kaplan Meyer disease free survival curve of mice of indicated genotypes. n represents the number of animals for each genotype. (**d**) PCR on genomic DNA prepared from tail snips of indicated genotypes to detect the *ROSA26*, *Act-Flpe*, the unrecombined ^*FSF*^*TGFβ*^*CA*^, and the recombined ^*FSF*^*TGFβ*^*CA*^ alleles. (**e**) Quantification of *TGFβ*^*CA*^ (left panel) and *eYFP* (right panel) mRNA by RT-PCR on total RNA prepared from [^FSF^TGFβ^CA^] and [Act-Flpe; ^FSF^TGFβ^CA^] ear skin samples. (**f**) Western blot analysis of eYFP and β-tubulin on whole protein extracts prepared from skin samples of indicated genotypes. In e and f, one representative experiment performed 3 times with skin samples from different mice is presented. In c and e, Prism 7.0 (Graphpad) was used to create graphs. In e, the error bars represent the standard deviation from technical duplicates.

In order to assess the activity of the *TGFβ*^*CA*^ transgene *in vivo*, we developed a strategy aiming at expressing the transgene at an early stage during embryonic development in all cell lineages. To that end, we bred [^FSF^TGFβ^CA^] mice with [Act-Flpe] mice (expressing the Flpe recombinase in all cell lineages from the blastocyst stage of development)^22^ to generate [Act-Flpe; ^FSF^TGFβ^CA^] compound individuals (Fig. 2a). Since TGFβ plays a crucial role in both development and adult homeostasis, it was expected that early-stage ubiquitous *TGFβ*^*CA*^ expression would result in notable deleterious effects. From 42 litters, 231 individuals were obtained (Fig. 2b). Among the four expected genotypes in the offspring, [Act-Flpe; ^FSF^TGFβ^CA^] individuals were significantly underrepresented in comparison with [WT], [Act-Flpe], [^FSF^TGFβ^CA^] individuals. From the 21 [Act-Flpe; ^FSF^TGFβ^CA^] mice genotyped after birth, 5 had died close to or at birth. Thus, at weaning, only 16 [Act-Flpe; ^FSF^TGFβ^CA^] mice were alive and 2/3 of the expected [Act-Flpe; ^FSF^TGFβ^CA^] mice were missing. [Act-Flpe; ^FSF^TGFβ^CA^] survivors did not present any obvious external defects, even though 5 mice were sacrificed beyond the age of one year (Fig. 2c and sup Table 1). We verified by PCR on genomic DNA prepared from tail samples that the excision of the STOP cassette was detectable (Recombined ^*FSF*^*TGFβ*^*CA*^ transgene) in these surviving mice (Fig. 2d). A significant increase in the expression of both *TGFβ*^*CA*^ (Fig. 2e, left panel) and *eYFP* (Fig. 2e, right panel) mRNA were observed *ex vivo* by RT-PCR performed on total RNA prepared from [Act-Flpe; ^FSF^TGFβ^CA^] mice ear skin samples compared to [^FSF^TGFβ^CA^] mice. Western blot performed on whole protein extracts from skin revealed the presence of the eYFP protein only in extracts prepared from [Act-Flpe; ^FSF^TGFβ^CA^] mice (Fig. 2f). Altogether these data demonstrate the robustness of this mouse model.

**Figure 3:**
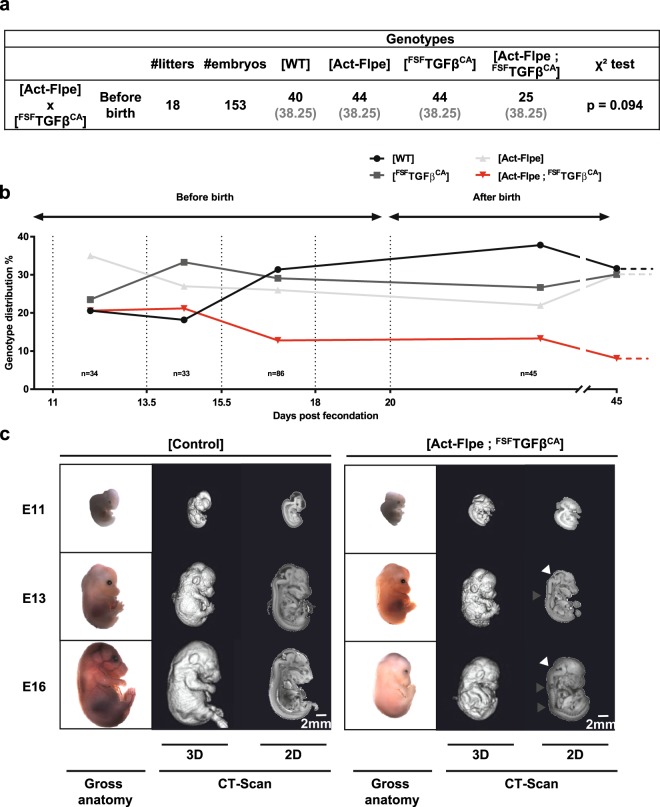
Developmental defects in [Act-Flpe; ^FSF^TGFβ^CA^] embryos. (**a**) [^FSF^TGFβ^CA^] were crossed with [Act-Flpe] mice. Total numbers (black) and expected numbers (grey) of litters, embryos, and offspring genotype distribution are presented. A χ² test was performed to test the “loss” of a significant proportion of [Act-Flpe; ^FSF^TGFβ^CA^] individuals just before birth. The Chi2 were calculated using Excel workbook developed by Montoliu^36^. (**b**) Distribution of the four expected genotypes in the offspring of [Act-Flpe] x [^FSF^TGFβ^CA^] at [E11-E13], [E14-E15], [E16-E18] and after birth. (**b**) Representative embryos of indicated genotypes at different stages of development (E11, E13, E16) visualized under the binocular (gross anatomy) and medial sagittal section by CT-Scan (computerized tomography scanner); 3D, three dimensions; 2D, two dimensions.

To understand the reasons underlying the lack of [Act-Flpe; ^FSF^TGFβ^CA^] individuals at weaning, we generated 153 embryos at different stages between E11.5 and E18 (Fig. 3a). We confirmed that a proportion (p = 0.094) of [Act-Flpe; ^FSF^TGFβ^CA^] embryos was missing prior to birth. Hence, we precisely analyzed embryos at different developmental stages and observed that approximately half of the [Act-Flpe; ^FSF^TGFβ^CA^] embryos had died by the E18 stage, according to the expected Mendelian proportions (Fig. 3b). We also observed macroscopically (gross anatomy) and by CT-scan (computerized tomography) scanner imaging, that half of the [Act-Flpe; ^FSF^TGFβ^CA^] embryos presented a severe growth retardation at the different stages (Fig. 3c). Brain (white arrowhead) and spinal column (grey arrowhead) defects were observed by CT-scan at stages E13 and E16. Altogether, these data demonstrate that the *TGFβ*^*CA*^ transgene can be conditionally expressed in the presence of Flp recombinase, and that the TGFβ^CA^ protein is functional *in vivo* as attested by i- the missing of a proportion of [Act-Flpe; ^FSF^TGFβ^CA^] late embryos and adults, and ii- the severe growth retardation observed in many [Act-Flpe; ^FSF^TGFβ^CA^] embryos.

**Figure 4:**
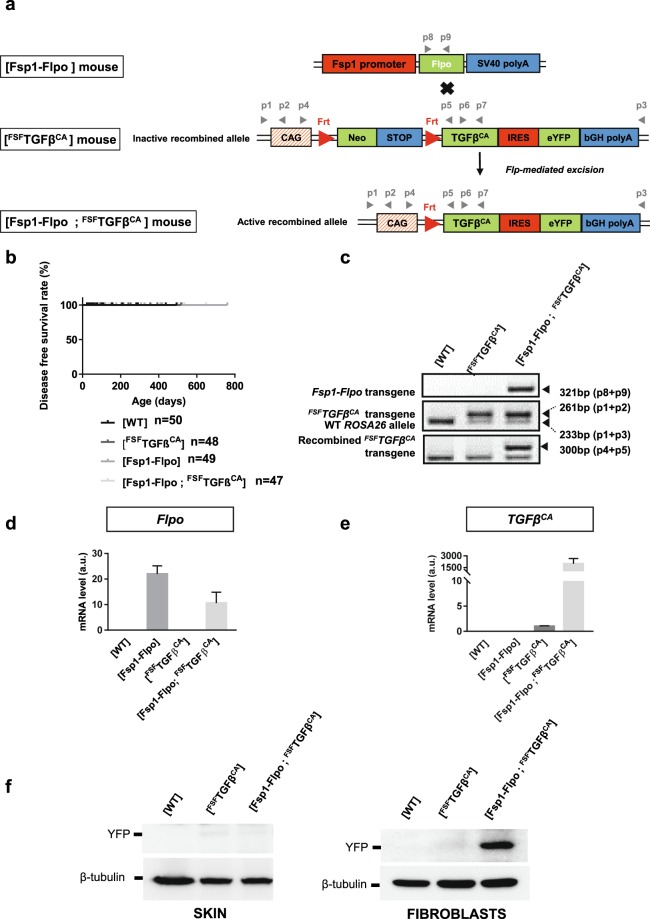
Generation of [Fsp1-Flpo; ^FSF^TGFβ^CA^] compound mice. (**a**) Breeding strategy ([Fsp1-Flpo] x [^FSF^TGFβ^CA^]) to generate [Fsp1-Flpo; ^FSF^TGFβ^CA^] individuals. Flp-mediated excision of the transcriptional “Stop” leading to *TGFβ*^*CA*^ expression is represented. Primers used for DNA genotyping (panel c), RT-PCR (panel d, e and Fig. 5b–d) are represented by grey arrowheads. CAG, composite constitutive human cytomegalovirus enhancer and chicken β-actin promoter; Neo, neomycin antibiotic resistance cassette; IRES, internal ribosome entry site; eYFP, enhanced yellow fluorescent protein; bGH polyA, bovine growth hormone polyadenylation signal. Fsp1, Fibroblast specific promoter 1; Flpo, Flpo recombinase; SV40, Simian Virus 40; polyA, polyadenylation signal. (**b**) Kaplan Meyer disease free survival curve of mice of indicated genotypes. n represents the number of animals for each genotype. (**c**) PCR on genomic DNA prepared from tail snips of indicated genotypes to detect the *ROSA26*, *Fsp1-Flpo*, the unrecombined ^*FSF*^*TGFβ*^*CA*^, and the recombined ^*FSF*^*TGFβ*^*CA*^ alleles. (**d**) Detection of *Flpo* mRNA by RT-PCR performed on total mRNA prepared from ear skin immortalized fibroblasts of indicated genotypes. (**e**) Detection of *TGFβ*^*CA*^ mRNA by RT-PCR performed on total mRNA prepared from ear skin immortalized fibroblasts of indicated genotypes. (**f)** Western blot analysis of eYFP and β-tubulin on whole protein extracts prepared from skin samples (left panel) and immortalized fibroblasts extracted from skin (right panel) of indicated genotypes. In (**c**–**f**) one representative experiment performed 3 times with biological material from different mice is presented. In b, d and e, Prism 7.0 (Graphpad) was used to create graphs. In d and e, the error bars represent the standard deviation from technical duplicates.

[Fsp1-Flpo] mice were crossed with [^FSF^TGFβ^CA^] mice to generate [Fsp1-Flpo; ^FSF^TGFβ^CA^] individuals (Fig. 4a). [Fsp1-Flpo; ^FSF^TGFβ^CA^] mice were obtained at the expected Mendelian *ratio* and did not present obvious phenotypic alterations. Some mice lived up to 2 years, similarly to control mice (Fig. 4b and sup Table 1). Recombination was verified on tail biopsies by PCR (Fig. 4c). As expected, RT-PCR revealed that Flpo was detected in immortalized ear skin fibroblasts prepared from [Fsp1-Flpo] and [Fsp1-Flpo; ^FSF^TGFβ^CA^] individuals (undetectable expression in [WT] and [^FSF^TGFβ^CA^] fibroblasts) (Fig. 4d). *TGFβ*^*CA*^ mRNA was undetectable in [WT] and [Fsp1-Flpo] cells, barely detectable in [^FSF^TGFβ^CA^] cells and highly expressed in [Fsp1-Flpo; ^FSF^TGFβ^CA^] cells (Fig. 4e). eYFP was not detected by Western blot analysis performed on whole protein extracts from skin (Fig. 4f, left panel) but on extracts from immortalized fibroblasts isolated from the skin of [Fsp1-Flpo; ^FSF^TGFβ^CA^] mice eYFP was detected (Fig. 4f, right panel).

**Figure 5:**
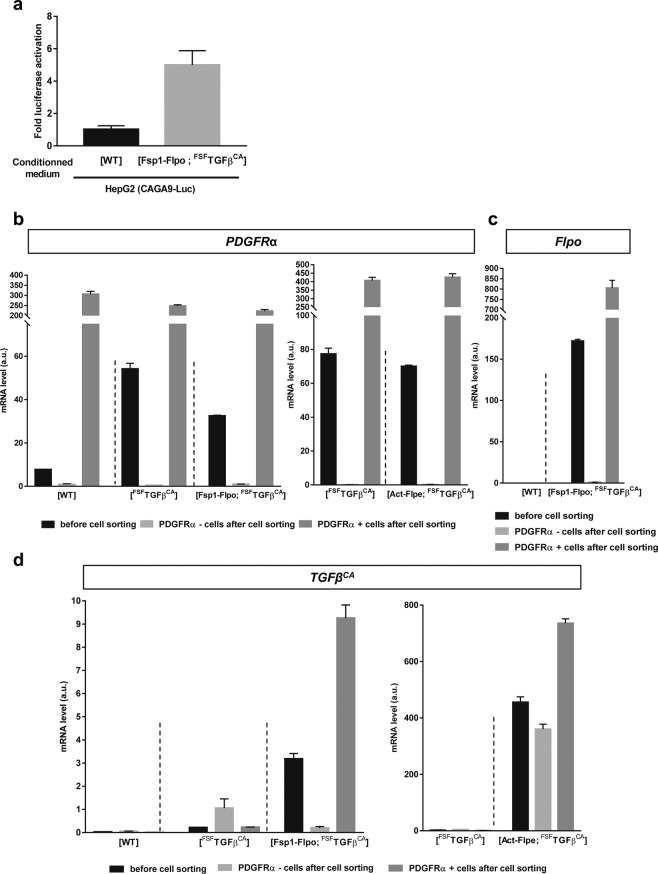
Functional validation of [Fsp1-Flpo; ^FSF^TGFβ^CA^] mice. (**a**) Reporter assay on HepG2 cells transiently transfected with a TGFβ-sensitive luciferase reporter plasmid (CAGA9-Luc) and cultured with the conditioned medium (CM) of immortalized skin fibroblasts from the indicated genotypes. (**b**–**d**) Quantification of *PDGFRα* (**b**), *Flpo* (**c**) and *TGFβ*^*CA*^ (**d**) mRNA by RT-PCR on total RNA prepared from cells with indicated genotypes present in back skin before and after cell sorting of PDGRα-positive cells. In (**a**–**d**), one representative experiment performed 3 times with biological material from different mice is presented. Prism 7.0 (Graphpad) was used to create graphs and the error bars represent the standard deviation from technical duplicates.

In order to determine whether the mutant TGFβ is efficiently secreted as an active form, we measured the activity of the cytokine in the conditioned medium (CM) of immortalized ear skin fibroblasts. To that end, we transiently transfected HepG2 cells with a TGFβ-sensitive luciferase reporter and observed that luciferase activity was significantly higher in cells cultured in the presence of CM from [Fsp1-Flpo; ^FSF^TGFβ^CA^] fibroblasts compared to [WT] (Fig. 5a). We isolated fibroblasts from [WT], [^FSF^TGFβ^CA^], [Fsp1-Flpo; ^FSF^TGFβ^CA^] and [Act-Flpe; ^FSF^TGFβ^CA^] mice back skin via cell sorting (Fig. 5b). [Act-Flpe; ^FSF^TGFβ^CA^] (ubiquitous expression of the transgene) mice were used as positive controls. RT-PCR confirmed the presence of PDGFRα mRNA in unsorted skin cells from all genotypes, and a clear enrichment after cell sorting using the PDGFRα antibody in PDGFRα-positive cells. Flpo recombinase was detected only in the skin of [Fsp1-Flpo; ^FSF^TGFβ^CA^] mice, and was clearly enriched in PDGFRα-positive cells after cell sorting (Fig. 5c). *TGFβ*^*CA*^ mRNA was detected in cells from [Fsp1-Flpo; ^FSF^TGFβ^CA^] mice and was enriched in PDGFRα-positive cells after cell sorting (Fig. 5d, left panel). As expected from the ubiquitous expression of the Flpe recombinase in [Act-Flpe; ^FSF^TGFβ^CA^] mice (not restricted to PDGRα-positive cells), TGFβ^CA^ was highly expressed in all cell lineages from [Act-Flpe; ^FSF^TGFβ^CA^] mice before and after cell sorting (Fig. 5d, right panel). Overall, these data confirm that [Fsp1-Flpo; ^FSF^TGFβ^CA^] mainly express TGFβ^CA^ in fibroblasts.

## Discussion

To explore *in vivo* the effect of increased local activity of TGFβ, we created a conditional transgenic mouse model (Flpo/Frt system) that expresses bioactive TGFβ in fibroblasts. We created a new mouse allele, *i.e*. the ^*FSF*^*TGFβ*^*CA*^ allele encoding a constitutionally active mutant form of TGFβ (*TGFβ*^*CA*^) under the control of a Frt-STOP-Frt (FSF) sequence, with the *Fsp1-Flpo* allele encoding the Flpo recombinase under the control of the *Fsp1* promoter. We validated the expression and the function of the ^*FSF*^*TGFβ*^*CA*^ allele by several *in vitro*, *ex vivo* and *in vivo* approaches. Particularly, we generated [Act-Flpe; ^FSF^TGFβ^CA^] individuals (expressing the transgene ubiquitously) and observed that a proportion of them was missing at weaning likely as a result of the severe growth retardation observed in many [Act-Flpe; ^FSF^TGFβ^CA^] embryos at different stages of development. The few [Act-Flpe; ^FSF^TGFβ^CA^] adult survivors looked normal. We next generated [Fsp1-Flpo; ^FSF^TGFβ^CA^] mice which presented no obvious phenotype either despite the fact that the ^*FSF*^*TGFβ*^*CA*^ allele was efficiently recombined and expressed in cells expressing the Flpo recombinase.

We were able to detect by RT-PCR very low levels of *TGFβ*^*CA*^ mRNA in the absence of Flp recombinase in [^FSF^TGFβ^CA^] mice. However, we demonstrated that this weak leak was not sufficient to induce phenotypical disorders, as attested by the absence of phenotype in [^FSF^TGFβ^CA^] mice, contrasting with the severe developmental defects we observed in [Actin-Flpe; ^FSF^TGFβ^CA^] embryos. Transgenic constitutively active TGFβ cannot be detected as the mutations do not occur in mature TGFβ^21^. Indeed, the mutations of cysteine residues 223 and 225 are present in the pro-domain thus preventing its interaction with mature TGFβ in a noncovalent manner to form a latent (inactive) complex. To circumvent this detection issue and be able to indirectly monitor the expression of the *TGFβ*^*CA*^ transgene, a sequence encoding a fluorescent reporter protein (eYFP) under the control of an IRES sequence was added to the transgene. *eYFP* mRNA was detected by RT-PCR in total RNA extracts from [Act-Flpe; ^FSF^TGFβ^CA^] mouse skin samples. We also detected the eYFP protein by Western blot analysis in whole protein extracts prepared from [Act-Flpe; ^FSF^TGFβ^CA^] mouse skin samples. Although we were unable to detect this protein in [Fsp1-Flpo; ^FSF^TGFβ^CA^] skin samples, Western blot analysis performed on immortalized fibroblasts prepared from [Fsp1-Flpo; ^FSF^TGFβ^CA^] mouse skin samples highlighted the protein. Of note, the eYFP protein was undetectable *in situ* both in cell culture and fixed or frozen tissues. Indeed, we were unable to visualize the eYFP protein, either directly (direct immunofluorescence) or indirectly (using a fluorescent or a biotinylated anti-YFP antibody). Even though we cannot rule out technical issues, the absence of detection likely results from a low level of protein expression due to a mechanism that remains to be determined (no translation or unstable protein for instance).

In order to validate the *in vivo* function of the *TGFβ*^*CA*^ transgene, we generated [Act-Flpe; ^FSF^TGFβ^CA^] individuals expressing TGFβ^CA^ in all cell lineages from the early embryonic stages. We observed that two thirds of these individuals were missing at weaning, and that most of the surviving embryos presented severe growth defects by the E15.5 stage. Such developmental retardations are not compatible with life after birth, likely explaining the absence of a great proportion of [Act-Flpe; ^FSF^TGFβ^CA^] individuals at weaning. The precise developmental defects at the origin of these abnormalities have not been addressed. However, we observed morphological features in accordance with an increased TGFβ signaling. Indeed, [Act-Flpe; ^FSF^TGFβ^CA^] embryos presented an abnormal forebrain development as described previously after injection of TGFβ in the brain of embryos^23^.

To express *TGFβ*^*CA*^ transgene in the fibroblasts, we generated [Fsp1-Flpo; ^FSF^TGFβ^CA^] individuals. *Fsp1* is expressed at a late stage of embryonic development preventing embryonic lethality^24–26^. We were able to observe the expression of Fsp1 protein in fibroblasts directly with an anti-Fsp1 antibody, and, *Fsp1* promoter activity with an *in vivo* reporter system ([Fsp1-Flpo; ^FSF^hPLAP]) (under review elsewhere). The *Fsp1* promoter has been successfully used by other groups to generate [Fsp1-Cre x TβRII^LoxP/LoxP^] mice carrying homozygous inactivation of type II TGFβ receptor in the stromal compartment^26,27^. These authors reported that abrogation of the canonical TGFβ signaling pathway in Fsp1-positive cells was compatible with full term embryonic development, though the males were sterile and both males and females died around 2 months of age, males developing preneoplastic lesions in the prostate epithelium and both sex developing squamous carcinoma of the forestomach. Fibroblast-specific activation of TGFβ signaling (constitutively active ligand overexpression or expression of a constitutively active receptor) has never been explored before and the present work is the first study to address this question, to our knowledge. We observed the absence of an obvious phenotype in [Fsp1-Flpo; ^FSF^TGFβ^CA^], contrasting with the phenotype observed in other models reporting an over-activation of TGFβ in specific tissues such as the mammary gland^6^ or head and neck epithelium^8^ and in other cell types such as hepatocytes^9^ or lung cells^10^. These observations may indicate that fibroblasts are not a sufficient source of bioactive TGFβ to induce defects observed in these models or may result from a transgene expression level too low to induce defects, or the presence of too few fibroblasts in organs in physiological conditions, or a lower bioactivity of the simian TGFβ used in this study compared to the porcine version used in other studies. Finally, and most likely, the [Fsp1-Flpo; ^FSF^TGFβ^CA^] model needs to be challenged with external or internal stresses to unveil a clear phenotype. For instance, it will be of particular interests to challenge these mice with ionizing radiations, pro-inflammatory stimuli or oncogenic chemicals or transgenes.

Cre-lox models do not easily permit independent temporal or spatial manipulation of different targeted genes. The combination of Flpo-Frt and Cre-loxP recombination systems or DRS bypasses this limitation^28,29^. Tissue-specific Flp drivers and Frt-flanked transgenes remain rare compared to tissue-specific Cre drivers and Floxed transgenes. Consequently, the development of DRS has been hindered. The Fsp1-[Flpo/^FSF^TGFβ^CA^] mouse model that we created in the present study, represents a significant contribution in the development of DRS in both fields of TGFβ signaling (^FSF^TGFβ^CA^) and microenvironment (Fsp1-Flpo). Indeed, our system paves the way for studies attempting to express bioactive TGFβ by fibroblasts (or another compartment if another specific Flp driver is used) in any Cre/lox-based model. Uncoupled spatiotemporal regulation of different genetic alterations using DRS should enable scientists to develop models better mimicking the complexity and heterogeneity of human diseases.

## Methods

### Biological material

#### Cells

HepG2 cells (ATCC® HB-8065™) were cultured in complete medium (Dulbecco’s Modified Eagle’s Medium, supplemented with 0.03% L-glutamine and containing 10% fetal bovine serum, a mix of 100 U mL^−1^ penicillin and 100 µg mL^−1^ streptomycin sulfate, 1% MEM non-essential amino acids) and propagated at 37 °C under 5% (v/v) CO_2_ atmosphere. Primary ear fibroblasts were isolated and cultured as follows: mouse ears were rinsed with 70% ethanol and samples of a few square-millimeters were harvested. Primary ear fibroblasts were isolated using mechanical dilacerations, followed by enzymatic dissociation (600 µL of a mix of collagenase D (4 mg mL^−1,^ COLLD-RO Roche) /Dispase II (4 mg mL^−1^, Roche) in RPMI medium) at 37 °C for 1 h. The reaction was stopped by adding 5 mL of complete medium (Dulbecco’s Modified Eagle’s Medium, supplemented with 0.03% L-glutamine and containing 10% fetal bovine serum, a mix of 100 U mL^−1^ penicillin and 100 µg mL^−1^ streptomycin sulfate) and cells were then incubated at 37 °C overnight. The following day, after filtration through a 100 µm pore cell strainer, cells were pelleted and reseeded in complete medium (Dulbecco’s Modified Eagle’s Medium, supplemented with 0.03% L-glutamine and containing 10% fetal bovine serum, a mix of 100 U mL^−1^ penicillin and 100 µg mL^−1^ streptomycin sulfate, 1% MEM non-essential amino acids and 50 µM β-mercaptoethanol). Medium was changed after 48 h. All cells were propagated at 37 °C under 5% (v/v) CO_2_ atmosphere. Immortalized fibroblasts were prepared as follows: primary fibroblasts were cultured to reach confluence in 100 mm cell culture dishes, split in two and grown in a 75 cm² flasks. At confluence, cells were divided, retaining only one third. This step was repeated until the cells entered into senescence. The medium was then changed every 2/3 days until immortalized clones grew. After immortalization, cells were cultured without β-mercaptoethanol. Sorted skin cells were prepared as follows: mice were sacrificed and shaved, and a piece of skin from the back of the animals was harvested and fat was removed. 5 mL of digestion medium (RPMI1640, 20% FBS, 1% PS, 1% Hepes, 1% Glutamine) was added to the skin in a petri dish. The skin was dilacerated using scissors and a cutter. Another 5 mL of digestion medium was added to help harvest the mixture. The mixture was then transferred to 50 mL tubes under agitation with a magnetic bar. 1 mL of collagenase type IA (10 mg/mL, C2674-1G (NA56)) and 200 µL of DNase1 (10 mg/mL, 11284932001 125,10) were added. The mix was agitated 30–90 min at 37 °C. The insoluble debris were eliminated by filtration onto a nylon grid. The digestion product was then centrifuged 5 min at 1,300 rpm and suspended in cell sorting buffer (1X PBS, 1% BSA, 0.5 mM EDTA). Cells were labelled in cell sorting buffer with PE anti-mouse CD140a/ PDGFRα antibody (BioLegend Cat. No. 135905 1:50) and PE Rat IgG2a, κ Isotype Control Antibody (BioLegend Cat. No. 400507 1:50). Cells were sorted using the BD FACS Aria III SORP (BECTON DICKINSON (BD™)).

#### Mice

The experiments were performed in compliance with the animal welfare guidelines of the European Union and with the French legislation. All experimental protocols (CECCAPP protocol #CLB-2012-012 #CLB-2017-007 #CLB-2018-025) were approved by the CECCAPP Région Rhône-Alpes ethical committee https://www.sfr-biosciences.fr/ethique/ceccapp/ceccapp under the control of the Ministère de l’Enseignement Supérieur et de la Recherche (#C2EA15) http://www.enseignementsup-recherche.gouv.fr/cid70597/l-utilisation-des-animaux-a-des-fins-scientifiques.html.

The ^*FSF*^*TGFβ*^*CA*^ allele was genetically engineered as follows by the ICS (Institut Clinique de la Souris, Strasbourg, France): we obtained the simian TGFβ^CA^ cDNA clone inside the πH3M plasmid^21^ from Pr. H. L. Moses, Vanderbilt-Ingram Cancer Center. We verified that the coding sequence contained the two point mutations C223S/C225S (data not shown). We functionally validated the *TGFβ*^*CA*^-expressing vector as attested by a 400-fold activation of the luciferase activity of a TGFβ-sensitive reporter gene transfected in HepG2 cells (see Supplementary Fig. S1). TGFβ^CA^ cDNA was further inserted into a targeting vector plasmid (see Supplementary Fig. S1) and fully sequenced (see Supplementary Fig. S2). [^FSF^TGFβ^CA^] mouse strain was generated by homologous recombination in ES cells after vector injection into C57BL/6 J blastocysts. 93 screened clones were injected and 3 positive clones (#51; #62; #77), containing the ^*FSF*^*TGFβ*^*CA*^ transgene inserted at the *ROSA26* locus, were identified by PCR on genomic DNA (see Supplementary Fig. S3**)**. Primers used for PCR are detailed in Table 1. At that point, 3 positive clones (#51; #62; #77) were validated by Southern blot with internal probes (see Supplementary Fig. S3). Digestions sites used to validate the 5′ and 3′ insertions are given in Table 2. 6 different digests are used to validate correct HR event. 3 digests validate the 5′ insertion, 3 other digests validate the 3′ insertion. Then, the 3 positive clones (#51; #62; #77) were validated by Southern blot with external probes (see Supplementary Fig. S3). Digestions used to validate with 5′ and 3′ probes are detailed in Table 2. One digest was used with 5′ probe and 3 different digests were used with 3′probe. After blastocyst injection, 2 out 3 ES clones (#51 and #62) gave rise to 8 chimeras (4 per ES clone). 2 heterozygous males and 2 heterozygous females were obtained after the breeding #51 chimera with C57BL/6 J mice, arguing in favor of an efficient germinal transmission of the transgene. The 2 heterozygous males were used for rederivation. Among the 9 pups obtained, 6 were positive for the transgene (2 males and 4 females) (see Supplementary Fig. S4). Primers used are positioned on gene sequence in Supplementary Fig. S3. These mice were then imported into our mouse facility (AniCan).

**Table 1:** List of primers.

	Primer Name/ alternate name	Primer sequences
*ROSA26* 5′HR arm flanking the ^*FSF*^*TGFβ*^*CA*^ transgene	Ef	GGTAGGGGATCGGGACTCTGGCGGG
	pCAG rev	GCAGAACGTGGGGCTCACCTCGACC
*ROSA26* 3′HR arm flanking the ^*FSF*^*TGFβ*^*CA*^ transgene	pGFP fw	GGCGACGTAAACGGCCACAAGTTCA
	Er	CTCAGTGGCTCAACAACACTTGGTC
^*FSF*^*TGFβ*^*CA*^ transgene	FWD / p1	AAAGTCGCTCTGAGTTGTTAT
	REV / p2	TGGGCTATGAACTAATGACCCCGTA
WT *ROSA26* allele	FWD / p1	AAAGTCGCTCTGAGTTGTTAT
	REV / p3	CCTTTAAGCCTGCCCAGAAG
Recombined ^*FSF*^*TGFβ*^*CA*^ transgene	FWD / p4	TAAGGGATCTGTAGGGCGCA
	REV/ p5	GTCTTGCAGGTGGATAGTCCT
^*FSF*^*TGFβ*^*CA*^ mRNA	FW / p6	CACAGCTCCTCTGACAGCAAA
	REV / p7	CGGGAGCTTTGCAGATGTTGG
*Flpo* transgene	FWD / p8	CTGGCCACATTCATCAACTGCGG
	REV / p9	CTTCTTCAGGGCCTTGTTGTAGCTG
*Flpo* mRNA	FWD / p8	CTGGCCACATTCATCAACTGCGG
	REV / p9	CTTCTTCAGGGCCTTGTTGTAGCTG
*Neo* cassette	FWD / p10	ATAGGAACTTCTAGGTCCCTCG
	REV / p11	TGCACGAGACTAGTGAGACG
*eYFP* transgene	FWD / p12	CCCGACAACCACTACCTGAG
	REV / p13	TTGTACAGCTCGTCCATGCC
*Act-Flpe* transgene	FWD	CACTGATATTGTAAGTAGTTTGC
	REV	CTAGTGCGAAGTAGTGATCAGG
*PDGFRα* mRNA	FWD	TTCAACGGAACCTTCAGCGT
	REV	ACGATCGTTTCTCCTGCCTT
*GAPDH* mRNA	FWD	CCCAGCAAGGACACTGAGCAAGAG
	REV	CTAGGCCCCTCCTGTTATTATGGGG

**Table 2:** Restriction enzymes sites on DNA.

Probe	Name	Genomic DNA digest	WT allele (kb)	Targeted Allele (kb)
Neo	5′ arm first digestion	Afl III	/	12.3
	5′ arm second digestion	Eco NI	/	11.7
	5′ arm third digestion	Eco RV		5.7
GFP	3′ arm first digestion	Kpn I	/	9
	3′ arm second digestion	Xba	/	7.7
	3′ arm third digestion	Pac I	/	10.9
5′ external	first digestion	Sex AI	6.7	14.7
3′ external	first digestion	Eco RI	15.6	13.8
	second digestion	Eco RV	11.5	13.8
	Third digestion	Hinc II	9.3	12.1

We generated the *Fsp1-Flpo* allele (in review elsewhere). Briefly, it is composed of a ~3,427 bp *Fsp1* promoter fragment (Exon_1/Intron_1/Partial_Exon_2; ENSMUSG00000001020) corresponding to the region previously described to drive the expression of Fsp1 in fibroblasts^25,26,30^. The *Flpo* transgene is a mouse codon-optimized Flp (Flpo) site-specific recombinase (SSR), which recombines DNA Frt-sites^31^.

The *Act-Flpe* allele was previously described^22^ and was obtained from the Jackson lab (B6;SJL-Tg(ACTFLPe)9205Dym/J, Stock No: 003800).

Mice were housed and bred in the “AniCan” specific pathogen-free animal facility of the CRCL (Centre de Recherche en Cancérologie de Lyon), France. The experiments were performed in compliance with the animal welfare guidelines of the European Union and with the French legislation (CECCAPP protocol #CLB-2012-012 #CLB-2017-007 #CLB-2018-025).

#### Embryos

Pregnant mice were killed by cervical dislocation. E11.5 to E18 embryos were removed from [Act-Flpe] or [^FSF^TGFβ^CA^] females previously impregnated by [^FSF^TGFβ^CA^] or [Act-Flpe] males, respectively, put on a 100 mm petri dishes on ice, and sacrificed by hypothermia in cold PBS. Neonates were decapitated. A piece of tail was sectioned for genotyping. Embryos were fixed 24 h in formalin and then stocked in PBS at 4 °C before use. Macroscopic images were acquired using SteREO Lumar.V12 coupled with AxioCam MRc5 (Zeiss).

For CT (computerized tomography)-scan analysis, formalin-fixed embryos were incubated 24 h under agitation in rotation at room temperature in 0.0125 M lugol (Sigma) (renewed every 8 h), then included in 1% agarose in a 11 mm in diameter centrifuge tube (Beckman coulter). Tubes with embryos in agarose were scanned (55kVp 181 µA, exposure time: 12.25 s, 0.5 mm aluminum filter) using the Perkin Elmer Quantum FX®. Medial sagittal sections were obtained using a Caliper analyze (AnalyzeDirect). 3D reconstruction was performed using 3DViewer. Gimp was used for improving the quality of the pictures obtained.

### Cell biology

#### Cell transfection

At day 1,200,000 ear fibroblasts were plated in 12-well plates. At day 2, cells in 1 mL of complete medium (Dulbecco’s Modified Eagle’s Medium, supplemented with 0.03% L-glutamine and containing 10% fetal bovine serum, a mix of 100 U mL^−1^ penicillin and 100 µg mL^−1^ streptomycin sulfate, 1% MEM non-essential amino acids) were transfected with 100 µL of transfection mix. These mix were maintained for 20 min at room temperature before transfection (800 ng of pSICO-Flpo plasmid (Addgene, 24969; Tyler Jacks), 4 µL of Lipofectamine® 2000 Transfection Reagent (Invitrogen), Opti-MEM medium (Thermofisher) to 100 µL). Medium was changed 6 h after transfection and replaced by a complete medium (Dulbecco’s Modified Eagle’s Medium, supplemented with 0.03% L-glutamine and containing 10% fetal bovine serum, a mix of 100 U mL^−1^ penicillin and 100 µg mL^−1^ streptomycin sulfate, 1% MEM non-essential amino acids). 48 h after transfection, wells were rinsed with PBS. For gDNA extraction, cells were lysed 30 min at 95 °C using 100 µL of Lysis buffer (25 mM NaOH, 0.2 mM EDTA) and 100 µL of neutralization buffer was added to stop the reaction (40 mM Tris-HCl).

#### Conditioned medium production and concentration

For conditioned medium (CM) production from cell cultures of primary fibroblasts, 300,000 cells were seeded onto a 6-well culture plate in complete DMEM. Following overnight culture for cell attachment, the medium was replaced by DMEM containing 0.5% FBS. After 24 h of culture, conditioned media were centrifuged at 1,200 rpm and cell supernatants were harvested and stored at −80 °C.

#### Luciferase assay

To measure the activity of TGFβ produced by fibroblasts from [Fsp1-Flpo; ^FSF^TGFβ^CA^] mice, and test the functionality of the TGFβ^CA^ transgene, on day 1, 30,000 HepG2 cells were seeded onto a 12-well culture plate in complete medium (Dulbecco’s Modified Eagle’s Medium, supplemented with 0.03% L-glutamine and containing 10% fetal bovine serum, a mix of 100 U mL^−1^ penicillin and 100 µg mL^−1^ streptomycin sulfate, 1% MEM non-essential amino acids). On day 2, HepG2 cells were transfected with 500 ng CAGA9-Luc (pGL3 MLP CAGA^32^) and 10 ng renilla (phRL-CMV) plasmids. For the functionality test of TGFβ^CA^ transgenes, we also transfected 800 ng of empty vector (pCMV5B WT, derived from pCMV5B hTbR2 addgene)^33^ or TGFβ plasmid (pTGFβ^CA^ πH3M simianTGFbeta1 CA C223S/C225S, Harold L. Moses (Vanderbilt-Ingram Cancer Center, Nashville, TN, USA). On day 3, HepG2 cells were deprived 1 h at 37 °C using 0.5 mL MEM 0% FBS. We added 0.5 mL/well of conditioned medium and treated or not with TGFβ purchased from Abnova (10 ng/mL final concentration). On day 4, cells were rinsed twice with PBS 1X, lysed 20–30 min with 50 µL of Passive Lysis Buffer 1X (Promega) under agitation and centrifuged 12,500 rpm for 1 min at room temperature. 50 µL of Luciferin substrate (Promega) and 10 µL of supernatant were mixed in a 96-well white plate and Firefly activity was read using the microplate reader infinite M1000 pro (TECAN). 50 µL of StopandGlo® reagent (Promega) was added and the renilla activity was read using the microplate reader.

### Molecular biology

#### Genomic and recombination PCR

DNA extraction and PCR were performed as previously described^12^ by using Taq DNA polymerase (Life Technologies) and primers cited in Table 1.

#### RNA/DNA extraction

RNA extraction from sorted skin cells: cells from cell sorting where lysed and RNA extracted using RNeasy Micro Kit (Qiagen).

Total RNA was extracted from transfected cells and purified from cells using lysis buffer from RNeasy Mini Kit (Qiagen).

#### RNA extraction from organs

Total RNAs were extracted from organs using ultraturax to mix them in a home-made RNA extraction solution (Guanidine thiocyanate 5 M (Sigma G6639), Citrate de sodium 0.5 M pH 7.0, Lauryl sarcosine 10%, βmercaptoethanol 1%). Purification was performed using the RNeasy Mini Kit (Qiagen).

#### Reverse-transcription PCR

First-strand cDNAs were prepared using 250 ng of RNA and SuperScript II Reverse Transcriptase in the presence of random primers (Invitrogen)^34^. Semi-quantitative PCR on cDNA was performed as previously described^35^ and using an Applied Biosystems StepOnePlus Real-Time PCR System with MESA GREEN qPCR MasterMix Plus (Eurogentec). All the real-time values were averaged and compared using the threshold cycle (C_T_) method, in which the amount of target RNA (2^−ΔΔCT^) was normalized against the endogenous expression of *GAPDH* (glyceraldehyde-3-phosphate dehydrogenase) (ΔC_T_). The amount of target mRNA in control cells or tissues was arbitrarily set at 1.0. Primers used for PCR are listed in Table 1.

#### Western blots

For protein analysis, cells were washed once with cold phosphate-buffered saline (PBS), and lysed with RIPA buffer (50 mM Tris-HCl pH 7.4, 150 mM NaCl, 1 mM EDTA, 0.5% sodium deoxycholate, 0.1% SDS, 1% Nonidet) containing a protease and phosphatase inhibitor cocktail. For tissue samples (skin), the tissue was homogenized (by grinding) in RIPA buffer. After protein quantification, 100 μg of protein was used for total lysate samples. Samples were separated by SDS-polyacrylamide gel electrophoresis (SDS-PAGE) and detected by immunoblot (WB) using Amersham ECL Detection Reagent (GE Healthcare). We used anti-GFP to detect YFP protein (Cell Signaling #2956) and anti-β-tubulin (Sigma T5293) primary antibodies and anti-rabbit or anti-mouse HRP-coupled antibodies from Dako (#P0260 and #P0448, respectively).

## Supplementary information

Supplementary information

Supplementary information2

## Data Availability

All data generated or analyzed during this study are included in this published article (and its Supplementary Information files). The datasets generated during and/or analyzed during the current study are available from the corresponding author on reasonable request.
