# Supplementary material for: Generation of a conditional Flpo/FRT mouse model expressing constitutively active TGFβ in fibroblasts
Source: Sci Rep. 2020 Mar 3;10:3880. doi: 10.1038/s41598-020-60272-3 (PMC7054254; doi:10.1038/s41598-020-60272-3)
Supplement: Supplementary file 1 — Supplementary information [file 41598_2020_60272_MOESM1_ESM.pdf]

Title: Generation of a conditional Flpo/FRT mouse model expressing constitutively active TGFβ in fibroblasts

Authors:
Victoire Cardot-Ruffino, Véronique Chauvet, Cassandre Caligaris, Adrien Bertrand-Chapel, Nicolas Chuvin, Roxane M. Pommier, Ulrich Valcourt, David Vincent, Sylvie Martel, Sophie Aires, Bastien Kaniewski, Pierre Dubus, Philippe Cassier, Stéphanie Sentis, Laurent Bartholin

a b

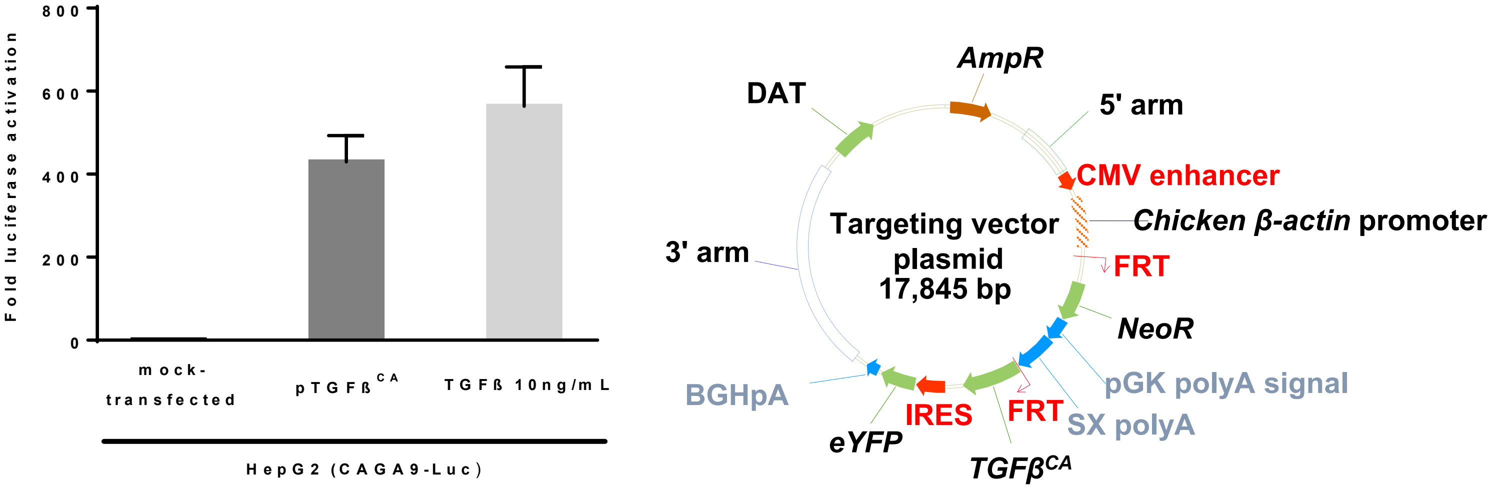

Fig. S1: Construction of the ^{FSF}TGFβ^{CA} targeting homologous recombination vector.

a) Luciferase assay in HepG2 cells transiently transfected with a TGFβ-sensitive reporter (CAGA9-luc) in the presence or not of TGFβ (positive control), and, ~~a~~ co-transfected with a plasmid expressing TGFβ^{CA} (pTGFβ^{CA}).
b) Circular map of the ^{FSF}TGFβ^{CA} transgenesis vector. DAT, Dopamine Transporter ; AmpR, ampicillin antibiotic resistance cassette; CMV enhancer, human cytomegalovirus enhancer and chicken β-actin promoter; NeoR, neomycin antibiotic resistance cassette; pGK, (phosphoglycerate kinase I) polyadenylation signal; TGFβ^{CA} sequence; IRES, internal ribosome entry site; eYFP, enhanced yellow fluorescent protein; bGH polyA, bovine growth hormone polyadenylation signal.

5' homology arm (1.1 kb)

CCGCGGCAGGCCCTCCGAGCGTGGTGGAGCCGTTCTGTGAGACAGCCGGGTACGAGTCGTGACGCTGGAAG
GGGCAAGCGGGTGGTGGGCAGGAATGCGGTCCGCCCTGCAGCAACCGGAGGGGGAGGGAGAAGGGAGCGG
AAAAGTCTCCACCGGACGCGGCCATGGCTCGGGGGGGGGGGGGCAGCGGAGGASCGCTTCCGGCCGACGTC
TCGTCGCTGATTGGCTTYTTTTCCTCCCGCCGTGTGTGAAAACACAAATGGCGTGTTTTGGTTGGCGTAAGGC
GCCTGTCAGTTAACGGCAGCCGGAGTGCGCAGCCGCCGGCAGCCTCGCTCTGCCCACTGGGTGGGGCGGGAG
GTAGGTGGGGTGAGGCGAGCTGNACGTGCGGGCGCGGTCGGCCTCTGGCGGGGCGGGGGAGGGGAGGGAG
GGTCAGCGAAAGTAGCTCGCGCGCGAGCGGCCGCCCACCCTCCCCTTCCTCTGGGGGAGTCGTTTTACCCGC
CGCCGGCCGGGCCTCGTCGTCTGATTGGCTCTCGGGGCCCAGAAAACTGGCCCTTGCCATTGGCTCGTGTTCG
TGCAAGTTGAGTCCATCCGCCGGCCAGCGGGGGCGGCGAGGAGGCGCTCCCAGGTTCCGGCCCTCCCCTCGG
CCCCGCGCCGCAGAGTCTGGCCGCGCGCCCCTGCGCAACGTGGCAGGAAGCGCGCGCTGGGGGCGGGGACG
GGCAGTAGGGCTGAGCGGCTGCGGGGCGGGTGCAAGCACGTTTCCGACTTGAGTTGCCTCAAGAGGGGCGT
GCTGAGCCAGACCTCCATCGCGCACTCCGGGGAGTGGAGGGAAGGAGCGAGGGCTCAGTTGGGCTGTTTTG
GAGGCAGGAAGCACTTGCTCTCCC**AAAGTCGCTCTGAGTTGTTAT**CAGTAAGGGAGCTGCAGTGGAGTAG
GCGGGGAGAAGGCCGCACCCTTCTCCGGAGGGGGGAGGGGAGTGTTGCAATACCTTTCTGGGAGTTCTCTGC
TGCCTCCTGGCTTCTGAGGACCGCCCTGGGCCTGGGAGAATCCCTTCCCCCTCTTCCCTCGTGATCTGCAACT
CCAGTCTTT

CMV Enhancer/Chicken β-actin promoter

ctagccttaattaactagttattaatagtaatcaattacggggtcattagttcatagcccatatatggagttccgcgttacataacttacggtaaatggcccgcctggctgaccgcccaac
gacccccgcccattgacgtcaataatgacgtatgttcccatagtaacgccaatagggactttccattgacgtcaatgggtggagtatttacggtaaactgcccacttggcagtacatca
agtgtatcatatgccaagtacgccccctattgacgtcaatgacggtaaatggcccgcctggcattatgcccagtacatgaccttatgggactttcctacttggcagtacatctacgtatt
agtcatcgctattaccatggtcgaggtgagccccacgttctgcttcactctccccatctcccccccctccccacccccaattttgtatttatttattttttaattattttgtgcagcgatgggg
gcggggggggggggggcgcgcgccaggcggggcggggcggggcgaggggcggggcggggcgaggcggagaggtgcggcggcagccaatcagagcggcgcgctcc
gaaagtttccttttatggcgaggcggcggcggcggcggccctataaaaagcgaagcgcgcggcgggcgggagtcgctgcgttgccttcgccccgtgccccgctccgccgccg
cctcgcgccgcccgccccggctctgactgaccgcgttactcccacaggtgagcgggcgggacggcccttctcctccgggctgtaattagcgcttggtttaatgacggcttgtttcttt
tctgtggctgcgtgaaagccttgaggggctccgggagggccctttgtgcggggggagcggctcggggggtgcgtgcgtgtgtgtgtgcgtggggagcgccgcgtgcggctcc
gcgctgcccggcggctgtgagcgctgcgggagcggcgcggggctttgtgcgctccgctgtgcgcgaggggagcgcggccgggggcggtgccccgcggtgcggggggggct
gcgaggggaacaaaggctgcgtgcggggtgtgtgcgtgggggggtgagcagggggtgtgggcgcggcggtcgggctgtaacccccccctgcacccccctccccgagttgct
gagcacggcccggcttcgggtgcggggctccgtacggggcgtggcgcggggctcgccgtgccgggcggggggtggcggcaggtgggggtgccgggcggggcggggcc
gcctcgggccggggagggctcgggggaggggcgcggcggcccccggagcgccggcggctgtcgaggcgcggcgagccgcagccattgccttttatggtaatcgtgcgaga
gggcgcagggacttcctttgtcccaaatctgtgcggagccgaaatctgggaggcgccgccgcaccccctctagcgggcgcggggcgaagcggtgcggcgccggcaggaag
gaaatgggcggggagggccttcgtgcgtcgccgcgccgccgtccccttctccctctccagcctcggggctgtccgcgggggggacggctgccttcgggggggacggggcagg
gcggggttcggcttctggcgtgtgaccggcggctcttaattaagggatctgtagggcgcagtagtccagggtttccttgatgatgtcatacttatcctgtcccttttttttccacagctcg
cggttgaggacaaactcttcgcggtctttccagtggggatcgacggtatcgtagagtcgaggccgctctagaactagtggatccggaacccttaat

FRT-Neo region FRT (2704pb = 2,7kb))

gaagttcctattctctagaaagt**ataggaacttctaggtccctcgacctgcaggaattctaccgggtaggggaggcgcttttcccaaggcagtctggagcatgcgctttagca**
gccccgctgggcacttggcgctacacaagtggcctctggcctcgcacacattccacatccaccggtaggcgccaaccggctccgttctttggtggccccttcgcgccacc
ttctactcctcccctagtcaggaagttcccccccgccccgcagctcgcgtcgtgcaggacgtgacaaatggaagtagcacgtctcactagtctcgtgcagatggacagca
ccgctgagcaatggaagcgggtaggcctttggggcagcggccaatagcagctttgctccttcgctttctgggctcagaggctgggaaggggtgggtccgggggcgggctcagg
ggcgggctcaggggcggggcgggcgcccgaaggtcctccggaggcccggcattctcgcacgcttcaaaagcgcacgtctgccgcgctgttctcctcttcctcatctccgggcct
ttcgacc**tgcagccaatatgggatcggccattgaacaagatggattgcacgcaggttctccggccgcttgggtggagaggctattcggctatgactgggcacaacagac**
aatcggctgctctgatgccgccgtgttccggctgtcagcgcaggggcgcccggttctttttgtcaagaccgacctgtccggtgccctgaatgaactgcaggacgaggcagcgcg
gctatcgtggctggccacgacgggcgttccttgcgcagctgtgctcgacgttgtcactgaagcgggaagggactggctgctattgggcgaagtgccggggcaggatctcctgtca
tctcaccttgctcctgccgagaaagtatccatcatggctgatgcaatgcggcggctgcatacgcttgatccggctacctgcccattcgaccaccaagcgaaacatcgcatcgagcg
agcacgtactcggatggaagccggtcttgtcgatcaggatgatctggacgaagagcatcaggggctcgcgccagccgaactgttcgccaggctcaaggcgcgcatgcccgacg
gcgatgatctcgtcgtgacccatggcgatgcctgcttgccgaatatcatggtggaaaatggccgcttttctggattcatcgactgtggccggctgggtgtggcggaccgctatcagg
acatagcgttggctacccgtgatattgctgaagagcttggcggcgaatgggctgaccgcttcctcgtgctttacggtatcgccgctcccgattcgcagcgcatcgccttctatcgcctt
cttgacgagttcttctgaggggatccgctgtaagtctgcagaaattgatgatctattaaacaataaagatgtccactaaaatggaagtttttcctgtcatactttgttaagaagggtgaga
acagagtacctacattttgaatggaaggattggagctacgggggtgggggtggggtgggattagataaatgcctgctctttactgaaggctctttactattgctttatgataatgtttcat
agttggatatcataatttaaacaagcaaaaccaaattaagggccagctcattcctcccactcatgatctatagatctatagatctctcgtgggatcattgtttttctcttgattcccactttgt
ggttctaagtactgtggtttccaaatgtgtcagtttcatagcctgaagaacgagatcagcagcctctgttccacatacacttcattctcagtattgttttgccaagttctaattccatcagaa
gcttgcagatctgcgactctagaggatctgcgactctagaggatcataatcagccataccacatttgtagaggttttacttgctttaaaaaacctcccacacctccccctgaacctgaa
acataaaatgaatgcaattgttgttgttaacttgtttattgcagcttataatggttacaaataaagcaatagcatcacaaatttcacaaataaagcatttttttcactgcattctagttgtggttt
gtccaaactcat

caatgtatcttatcatgtctggatctgcgactctagaggatcataatcagccataccacatttgtagaggttttacttgctttaaaaaacctcccacacctccccctgaacctgaaacata
aaatgaatgcaattgttgttgttaacttgtttattgcagcttataatggttacaaataaagcaatagcatcacaaatttcacaaataaagcatttttttcactgcattctagttgtggtttgtcca
aactcatcaatgtatcttatcatgtctggatctgcgactctagaggatcataatcagccataccacatttgtagaggttttacttgctttaaaaaacctcccacacctccccctgaacctga
aacataaaatgaatgcaattgttgttgttaacttgtttattgcagcttataatggttacaaataaagcaatagcatcacaaatttcacaaataaagcatttttttcactgcattctagttgtggtt
tgtccaaactcatcaatgtatcttatcatgtctggatccccatcaagctgatccggaacccttaatgaagttcctattctctagaaagtataggaacttc

TGFBmut/ IRES EYFP BGHpA

tagagctagcgcctccccATGCCGCCCTCCGGGCTGCGGCTGCTGCCGCTGCTGCTACCGCTGCTGTGGCTACTGGTGC
TGACGCCTAGCCGGCCGGCCGC**AGGACTATCCACCTGCAAGAC**TATCGACATGGAGCTGGTGAAGCGGAA
GCGCATCGAGACCATCCGCGGCCAGATCCTGTCCAAGCTGCGGCTCGCCAGCCCCCCGAGCCAGGGGGAGGT
GCCGCCCGGCCCGCTGCCCGAGGCCGTGCTCGCCCTGTACAACAGCACCCGCGACCGGGTGGCCGGGGAGA
GTGCGGAGCCGGAGCCCGAACCGGAGGCCGACTACTACGCCAAGGAGGTCACCCGCGTGCTAATGGTGGAA
ACCCACAACGAAATCTATGACAAGTTCAAGCAGAGCACACACAGCATATATATGTTCTTCAACACATCAGAG
CTCCGAGAAGCAGTACCTGAACCTGTGTTGCTCTCCCGGGCAGAGCTGCGTCTGCTGAGGCTCAAGTTAAAA
GTGGAGCAGCATGTGGAGCTGTACCAGAAATACAGCAACAATTCCTGGCGATACCTCAGCAACCGGCTGCTG
GCGCCCAGCAACTCGCCGGAGTGGTTGTCTTTTGATGTCACCGGAGTTGTGCGGCAGTGGTTGAGCCGCGGA
GGGGAAATTGAGGGCTTTCGCCTTAGCGCC**CAC**A**GCTCCT**C**TGACAGCAAAGATAACA**CACTGCAAGTGG
ACATCAACGGGTTCACTACCGGCCGCCGAGGTGACCTGGCCACAATTCATGGCATGAACCGGCCTTTCCTGC
TTCTCATGGCCACCCCACTGGAGAGGGC**CCAACATCTGCAAAGCTCCCG**GCACCGCCGAGCCCTGGACACC
AACTACTGCTTCAGCTCCACGGAGAAGAACTGCTGCGTGCGGCAGCTGTATATTGACTTCCGCAAGGACCTC
GGCTGGAAGTGGATCCACGAGCCCAAGGGCTACCATGCCAACTTCTGCCTGGGGCCCTGTCCCTACATTTGG
AGCCTGGACACGCAGTACAGCAAGGTCCTGGCCCTGTACAACCAGCATAACCCGGGCGCCTCGGCGGCGCC
GTGCTGCGTGCCGCAGGCGCTGGAGCCACTGCCCATCGTGTACTACGTGGGCCGCAAGCCCAAGGTGGAGCA
GCTGTCCAACATGATCGTGCGCTCCTGCAAATGCAGCtgagaattcagtggatccactagtaacggccgccagtgtgctggaattaattcgctgt
ctgcgagggccagctgttggggtgagtactccctctcaaaagcgggcatgacttctgcgctaagattgtcagtttccaaaaacgaggaggatttgatattcacctggcccgcggtga
tgcctttgagggtggccgcgtccatctggtcagaaaagacaatctttttgttgtcaagcttgaggtgtggcaggcttgagatctggccatacacttgagtgacaatgacatccactttgc
ctttctctccacaggtgtccactcccaggtccaactgcaggtcgagcatgcatctagggcggccaattccgcccctctccctcccccccccctaacgttactggccgaagccgcttg
gaataaggccggtgtgcgtttgtctatatgttattttccaccatattgccgtcttttggcaatgtgagggcccggaaacctggccctgtcttcttgacgagcattcctaggggtctttcccc
tctcgccaaaggaatgcaaggtctgttgaatgtcgtgaaggaagcagttcctctggaagcttcttgaagacaaacaacgtctgtagcgaccctttgcaggcagcggaaccccccac
ctggcgacaggtgcctctgcggccaaaagccacgtgtataagatacacctgcaaaggcggcacaaccccagtgccacgttgtgagttggatagttgtggaaagagtcaaatggct
ctcctcaagcgtattcaacaaggggctgaaggatgcccagaaggtaccccattgtatgggatctgatctggggcctcggtgcacatgctttacatgtgtttagtcgaggttaaaaaa
cgtctaggccccccgaaccacggggacgtggttttcctttgaaaaacacgatgataagcttgccacaacccgggatccaccggtcgccaccatggtgagcaagggcgaggagc
tgttcaccggggtggtgcccatcctggtcgagctggacggcgacgtaaacggccacaagttcagcgtgtccggcgagggcgagggcgatgccacctacggcaagctgaccct
gaagttcatctgcaccaccggcaagctgcccgtgccctggcccaccctcgtgaccaccttcggctacggcctgcagtgcttcgcccgctaccccgaccacatgaagcagcacga
cttcttcaagtccgccatgcccgaaggctacgtccaggagcgcaccatcttcttcaaggacgacggcaactacaagacccgcgccgaggtgatagtcgagggcgacaccctggt
gaaccgcatcgagctgaagggcatcgacttcaaggaggacggcaacatcctggggcacaagctggagtacaactacaacagccacaacgtctatatcatggccgacaagcag
aagaacggcatcaaggtgaacttcaagatccgccacaacatcgaggacggcagcgtgcagctcgccgaccactaccagcagaacacccccatcggcgacggccccgtgctg
ctgcccgacaaccactacctgagctaccagtccgccctgagcaaagaccccaacgagaagcgcgatcacatggtcctgctggagttcgtgaccgccgccgggatcactctcgg
catggacgagctgtacaagtaaagcggccctagagctcgctgatcagcctcgactgtgccttctagttgccagccatctgttgtttgcccctcccccgtgccttccttgaccctggaa
ggtgccactcccactgtcctttcctaataaaatgaggaaattgcatcgcattgtctgagtaggtgtcattctattctggggggtggggtggggcaggacagcaagggggaggattgg
gaagacaatagcaggcatgctggggatgcggtgggctctatggcttctgaggcggaaagaaccagctggggctcgagggggcccccgcgggcggccgcgagctcgctgatca
gcctcgactgtgccttctagttgccagccatctgttgtttgcccctcccccgtgccttccttgaccctggaaggtgccactcccactgtcctttcctaataaaatgaggaaattgcatcgc
attgtctgagtaggtgtcattctattctggggggtggggtggggcaggacagcaagggggaggattgggaagacaatagcaggcatgctggggatgcggtgggctctatggcttc
tgaggcggaaagaaccagctggggctcgatcctctagttggcgcgccggctagaa

3’ Homology arm (4.2kb)

GATGGGCGGGAGT**CTTCTGGGCAGGCTTAAAGG**CTAACCTGGTGTGTGGGCGTTGTCCTGCAGGGGAATTGAACAGGTGTAAAATTG
GAGGGACAAGACTTCCCACAGATTTTCGGTTTTGTCGGGAAGTTTTTTAATAGGGGCAAATAGGAAAATGGAGGATAGGAGTCATCTG
GGGTTTATGCAGCAAAACTACAGGTATATTGCTTGTATCCGCCTCGGAGATTTCCATGAGGAGATAAAGACATGTCACCCGAGTTTATA
CTCTCCTGCTTAGATCCTACTACAGTATGAAATACAGTGTYGCGAGGTAGACTATGTAAGCAGATTTAATCATTTTAAAGAGCCCAGTAC
TTCATATCCATTTCTCCCGCTCCTTCTGCAGCCTTATCAAAAGGTATTTAGAACACTCATTTTAGCCCCATTTTCATTTATTATACTGGCTT
ATCCAACCCCTAGACAGAGCATTGGCATTTTCCCTTTCCTGATCTTAGAAGTCTGATGACTCATGAAACCAGACAGATTAGTTACATACA
CCACAAATCGAGGCTGTAGCTGGGGCCTCAACACTGCAGTTCTTTTATAACTCCTTAGTACACTTTTTGTTGATCCTTTGCCTTGATCCTT
AATTTTCAGTGTCTATCACCTCTCCCGT

CAGGTGGTGTTCCACATTTGGGCCTATTCTCAGTCCAGGGAGTTTACAACAATAGATGTATTGAGAATCCAA
CCTAAAGCTTAACTTTCCACTCCCATGAATGCCTCTCTCCTTTTTCTCCATTATAACTGAGCTATWACCATTAA
TGGTTTCAGGTGGATGTCTCCTCCCCCAATATACCTGATGTATCTACATATTGCCAGGCTGATATTTTAAGAC
ATWAAAGGTATATTTCATTATTGAGCCACATGGTATTGATTACTGCTACTAAAATTTTGTCATTGTACACATC
TGTAAAAGGTGGTTCCTTTTGGAATGCAAAGTTCAGGTGTTTGTTGTCTTTCCTGACCTAAGGTCTTGTGAGC
TTGTATTTTTTCTATTTAAGCAGTGCTTTCTCTTGGACTGGCTTGACTCATGGCATTCTACACGTTATTGCTGG
TCTAAATGTGATTTTGCCAAGCTTCTTCAGGACCTATAATTTTGCTTGACTTGTAGCCAAACACAAGTAAAAT
GATTAAGCAACAAATGTATTTGTGAAGCTTGGTTTTTAGGTTGTTGTGTTGTGTGTGCTTGTGCTCTATAATA
ATACTATCCAGGGGCTGGAGAGGTGGCTCGGAGTTCAAGAGCACAGACTGCTCTTCCAGAAGTCCTGAGTTC
AATTCCCAGCAACCACATGGTGGCTCACAACCATCTGTAATGGGATCTGATGCCCTCTTCTGGTGTGTCTGAA
GACCACAAGTGTATTCACATTAAATAAATAATCCTCCTTCTTCTTCTTTTTTTTTTTTTAAAGAGAATWCTGTC
TCCAGTAGAATTACTGAAGTAATGAAATACTTTGTGTTTGTTCCAATATGGWAGCCAATAATCAAATACTCT
TWAGCACTGGAAATGTACCAAGGAACTATTTTATTTAAGTGWACTGTGGACAGAGGAGCCATAACTGCAGA
CTTGTGGGATACAGAAGACCAATGCAGACTTAATGTCTTTTCTCTTACACTAAGCAATAAAGAAATAAAAAT
TGAACTTCTAGTATCCTATTTGTTAAACTGCTAGCTTTACTAACTTTTGTGCTTCATCTATACAAAGCTGAAAG
CTAAGTCTGCAGCCATTACTAAACATGAAAGCAAGTAATGATAATTTTGGATTTCAAAAATGTAGGGCCAGA
GTTTAGCCAGCCAGTGGTGGTGCTTGCCTTTATGCCTTAATCCCAGCACTCTGGAGGCAGAGACAGGCAGAT
CTCTGAGTTTGAGCCCAGCCTGGTCTACACATCAAGTTCTATCTAGGATAGCCAGGAATACACACAGAAACC
CTGTTGGGGAGGGGGGCTCTGAGATTTCATAAAATTATAATTGAAGCATTCCCTAATGAGCCACTATGGATG
TGGCTAAATCCGTCTACCTTTCTGATGAGATTTGGGTATTATTTTTTCTGTCTCTGCTGTTGGTTGGGTCTTTTG
ACACTGTGGGCTTTCTTAAAGCCTCCTTCCCTGCCATGTGGTCTCTTGTTTGCTACTAACTTCCCATGGCTTAA
ATGGCATGGCTTTTTGCCTTCTAAGGGCAGCTGCTGAGWTTTGCAGCCTGATTTCCAGGGTGGGGTTGGGAA
ATCTTTCAAACACTAAAATTGTCCTTTAATTTTTTTTTAAAAAATGGGTTATATAATAAACCTCATAAAATAG
TTATGAGGAGTGAGGTGGACTAATATTAATGAGTCCCTCCCCTATAAAAGAGCTATTAAGGCTTTTTGTCTTA
TACTAACTTTTTTTTTAAATGTGGTATCTTTAGAACCAAGGGTCTTAGAGTTTTAGTATACAGAAACTGTTGC
ATCGCTTAATCAGATTTTCTAGTTTCAAATCCAGAGAATCCAAATTCTTCACAGCCAAAGTCAAATTAAGAAT
TTCTGACTTTAATGTTATTTGCTACTGTGAATATAAAATGATAGCTTTTCCTGAGGCAGGGTCTCACTATGTAT
CTCTGCCTGATCTGCAACAAGATATGTAGACTAAAGTTCTGCCTGCTTTTGTCTCCTGAATACTAAGGTTAAA
ATGTAGTAATACTTTTGGAACTTGCAGGTCAGATTCTTTTATAGGGGACACACTAAGGGAGCTTGGGTGATA
GTTGGTAAATGTGTTTAAGTGATGAAAACTTGAATTATTATCACCGCAACCTACTTTTTAAAAAAAAAAGCC
AGGCCTGTTAGAGCATGCTAAGGGATCCCTAGGACTTGCTGAGCACACAAGAGTAGTACTTGGCAGGCTCCT
GGTGAGAGCATATTTCAAAAAACAAGGCAGACAACCAAGAAACTACAGTAAGGTTACCTGTCTTTAACCATC
TGCATATACACAGGGATATTAAAATATTCCAAATAATATTTCATTCAAGTTTTCCCCCATCAAATTGGGACAT
GGATTTCTCCGGTGAATAGGCAGAGTTGGAAACTAAACAAATGTTGGTTTTGTGATTTGTGAAATTGTTTTCA
AGTGATAGTTAAAGCCCATGAGATACAGAACAAAGCTGCTATTTCGAGGTCTCTTGGTTATACTCAGAAGCA
CTTCTTTGGGTTTCCCTGCACTATCCTGATCATGTGCTAGGCCTWCCTTAGGCTGATTGTTGTTCAAATAACTT
AAGTTTCCTGTCAGGTGATGTCATATGATTTCATATATCAAGGCAAAACATGTTATATATGTTAAACATTTGK
ACTTAATGTGAAAGTTAGGTCTTTGTGGGTTTTGATTTTAATTTCAAAACCTGAGCTAAATAAGTCATTTTAC
ATGTCTTACATTTGGTGAATTGTATATTGTGGTTTGCAGGCAAGACTCTCTGACCTAGTAACCCTCCTATAGA
GCACTTTGCTGGGTCACAAGTCTAGGAGTCAAGCATTTCACCTTGAAGTTGAGACGTTTTGTTAGTGTATACT
AGTTATATGTTGGAGGACATGTTTATCCAGAAGATATTCAGGACTATTTTTGACTGGGCTAAGGAATTGATTC
TGATTAGCACTGTTAGTGAGCATTGAGTGGCCTTTAGGCTTGAATTGGAGTCACTTGTATATCTCAAATAATG
CTGGCCTTTTTTWAAAAGCCCTTGTTCTTTATCACCCTGTTTTCTACATAATTTTTGTTCAAAGAAATACTTGT
TTGGATCTCCTTTTGACAACAATAGCATGTTTTCAAGCCATATTTTTTTTCCTTTTTTTTTTTTTTTTTGGTTTTT
CGAGACAGGGTTTCTCTGTATAGCCCTGGCTGTCCTGGAACTCACTTTGTAGACCAGGCTGGCCTCGAACTCA
GAAATCCGCCTGCCTCTGCCTCCTGAGTGCCGGGATTAAAGGCGTGCACCACCACGCCTGGCTAAGTTGGAT
ATTTTGTATATAACTATAACCAATACTAACTCCACTGGGTGGATTTTTAATTCAGTCAGTAGTCTTAAGTGGT
CTTTATTGGCCCTTATTAAAATCTACTGTTCACTCTAACAGAGGCTGTTGGACTAGTGGSACTAAGCAACTTC
CTACGGATATACTAGCAGATAAGGGTCAGGGATAGAAACTAGTCTAGCGTTTTGTATACCTACCAGCTTATA
CTACCTTGTTCTGAT

Vector backbone sequence

Agaaatatttaggacatctagcttatcgatccgtcgacggtatcgataagcttgatatcgaattctaccgggtaggggaggcgcttttccaaggcagtctgagcatgcgcttagcagc
cccgctggcacttggcgctacacaagtggcctytggcctcgcacacattccacatccaccggtaggcgccaaccggctccgttctttggtggccccttcgcgccaccttctwctcc
tc

ccctagtcaggaagttcccccccgccccgcagctcgcgtcgtsaggacgtgacaaatggaagtagcacgtctcactagtctcgtcagatggacagcaccgctgagcaatggaag
cgggtaggcctttggggcagcggccaatagcagctttgctccttcgctttctgggctcagaggctgggaaggggtgggtccgggggcgggctcaggggcgggctcaggggcg
gggcgggcgcccgaaggtcctccggaggcccggcattctgcacgcttcaaaagcgcacgtctgccgcgctgttctcctcttcctcatctccgggcctttcgacctgcaggtcctcg
ccatggatcctgatgatgttgttattcttctaatcttttgtatggaaaacttttcttcgtaccacgggactaaacctggttatgtagattccattcaaaaaggtatacaaaagccaaaatctgg
tacacaaggaaattatgacgatgattggaaagggttttatagtaccgacaataaatacgacgctgcgggatactctgtagataatgaaaacccgctctctggaaaagctggaggcgt
ggtcaaagtgacgtatccaggactgacgaaggttctcgcactaaaagtggataatgccgaaactattaagaaagagttaggtttaagtctcactgaaccgttgatggagcaagtcgg
aacggaagagtttatcaaaaggttcggtgatggtgcttcgcgtgtagtgctcagccttcccttcgctgaggggagttctagcgttgaatatattaataactgggaacaggcgaaagcg
ttaagcgtagaacttgagattaattttgaaacccgtggaaaacgtggccaagatgcgatgtatgagtatatggctcaagcctgtgcaggaaatcgtgtcaggcgatctctttgtgaag
gaaccttacttctgtggtgtgacataattggacaaactacctacagagatttaaagctctaaggtaaatataaaatttttaagtgtataatgtgttaaactactgattctaattgtttgtgtattt
tagattccaacctatggaactgatgaatgggagcagtggtggaatgcagatcctagagctcgctgatcagcctcgactgtgccttctagttgccagccatctgttgtttgcccctcccc
cgtgccttccttgaccctggaaggtgccactcccactgtcctttcctaataaaatgaggaaattgcatcgcattgtctgagtaggtgtcattctattctggggggtggggtggggcagg
acagcaagggggaggattgggaagacaatagcaggcatgctggggatgcggtgggctctatggcttctgaggcggaaagaaccagctggggctcgacctcgagggggggcc
cggtacccagcttttgttccctttagtgagggttaattgcgcgcttggcgtaatcatggtcatagctgtttcctgtgtgaaattgttatccgctcacaattccacacaacatacgagccgg
aagcataaagtgtaaagcctggggtgcctaatgagtgagctaactcacattaattgcgttgcgctcactgcccgctttccagtcgggaaacctgtcgtgccagctgcattaatgaatc
ggccaacgcgcggggagaggcggtttgcgtattgggcgctcttccgcttcctcgctcactgactcgctgcgctcggtcgttcggctgcggcgagcggtatcagctcactcaaagg
cggtaatacggttatccacagaatcaggggataacgcaggaaagaacatgtgagcaaaaggccagcaaaaggccaggaaccgtaaaaaggccgcgttgctggcgtttttccata
ggctccgcccccctgacgagcatcacaaaaatcgacgctcaagtcagaggtggcgaaacccgacaggactataaagataccaggcgtttccccctggaagctccctcgtgcgct
ctcctgttccgaccctgccgcttaccggatacctgtccgcctttctcccttcgggaagcgtggcgctttctcatagctcacgctgtaggtatctcagttcggtgtaggtcgttcgctccaa
gctgggctgtgtgcacgaaccccccgttcagcccgaccgctgcgccttatccggtaactatcgtcttgagtccaacccggtaagacacgacttatcgccactggcagcagccact
ggtaacaggattagcagagcgaggtatgtaggcggtgctacagagttcttgaagtggtggcctaactacggctacactagaaggacagtatttggtatctgcgctctgctgaagcca
gttaccttcggaaaaagagttggtagctcttgatccggcaaacaaaccaccgctggtagcggtggtttttttgtttgcaagcagcagattacgcgcagaaaaaaaggatctcaagaa
gatcctttgatcttttctacggggtctgacgctcagtggaacgaaaactcacgttaagggattttggtcatgagattatcaaaaaggatcttcacctagatccttttaaattaaaaatgaag
ttttaaatcaatctaaagtatatatgagtaaacttggtctgacagttaccaatgcttaatcagtgaggcacctatctcagcgatctgtctatttcgttcatccatagttgcctgactccccgtc
gtgtagataactacgatacgggagggcttaccatctggccccagtgctgcaatgataccgcgagacccacgctcaccggctccagatttatcagcaataaaccagccagccgga
agggccgagcgcagaagtggtcctgcaactttatccgcctccatccagtctattaattgttgccgggaagctagagtaagtagttcgccagttaatagtttgcgcaacgttgttgccatt
gctacaggcatcgtggtgtcacgctcgtcgtttggtatggcttcattcagctccggttcccaacgatcaaggcgagttacatgatcccccatgttgtgcaaaaaagcggttagctcctt
cggtcctccgatcgttgtcagaagtaagttggccgcagtgttatcactcatggttatggcagcactgcataattctcttactgtcatgccatccgtaagatgcttttctgtgactggtgagt
actcaaccaagtcattctgagaatagtgtatgcggcgaccgagttgctcttgcccggcgtcaatacgggataataccgcgccacatagcagaactttaaaagtgctcatcattggaa
aacgttcttcggggcgaaaactctcaaggatcttaccgctgttgagatccagttcgatgtaacccactcgtgcacccaactgatcttcagcatcttttactttcaccagcgtttctgggtg
agcaaaaacaggaaggcaaaatgccgcaaaaaagggaataagggcgacacggaaatgttgaatactcatactcttcctttttcaatattattgaagcatttatcagggttattgtctcat
gagcggatacatatttgaatgtatttagaaaaataaacaaataggggttccgcgcacatttccccgaaaagtgccacctaaattgtaagcgttaatattttgttaaaattcgcgttaaattt
ttgttaaatcagctcattttttaaccaataggccgaaatcggcaaaatcccttataaatcaaaagaatagaccgagatagggttgagtgttgttccagtttggaacaagagtccactatta
aagaacgtggactccaacgtcaaagggcgaaaaaccgtctatcagggcgatggcccactacgtgaaccatcaccctaatcaagttttttggggtcgaggtgccgtaaagcactaa
atcggaaccctaaagggagcccccgatttagagcttgacggggaaagccggcgaacgtggcgagaaaggaagggaagaaagcgaaaggagcgggcgctagggcgctggc
aagtgtagcggtcacgctgcgcgtaaccaccacacccgccgcgcttaatgcgccgctacagggcgcgtcccattcgccattcaggctgcgcaactgttgggaagggcgatcggt
gcgggcctcttcgctattacgccagctggcgaaagggggatgtgctgcaaggcgattaagttgggtaacgccagggttttcccagtcacgacgttgtaaaacgacggccagtgag
cgcgcgtaatacgactcactatagggcgaattggagctcc

Fig. S2: Verified sequence of the *$^{FSF}TGF\beta^{CA}$* transgene cloned inside the recombination vector.

Result of sequencing. 5' homology arm - CMV (human cytomegalovirus) Enhancer/Chicken β-actin promoter - FRT-neomycin antibiotic resistance cassette-FRT- TGFβ^{CA} sequence/IRES (internal ribosome entry site) - eYFP (enhanced yellow fluorescent protein) - BGHpA (bovine growth hormone polyadenylation signal) - 3' Homology arm, Vector backbone sequence. Primers sequences used for PCR and RT-PCR are underlined.

a

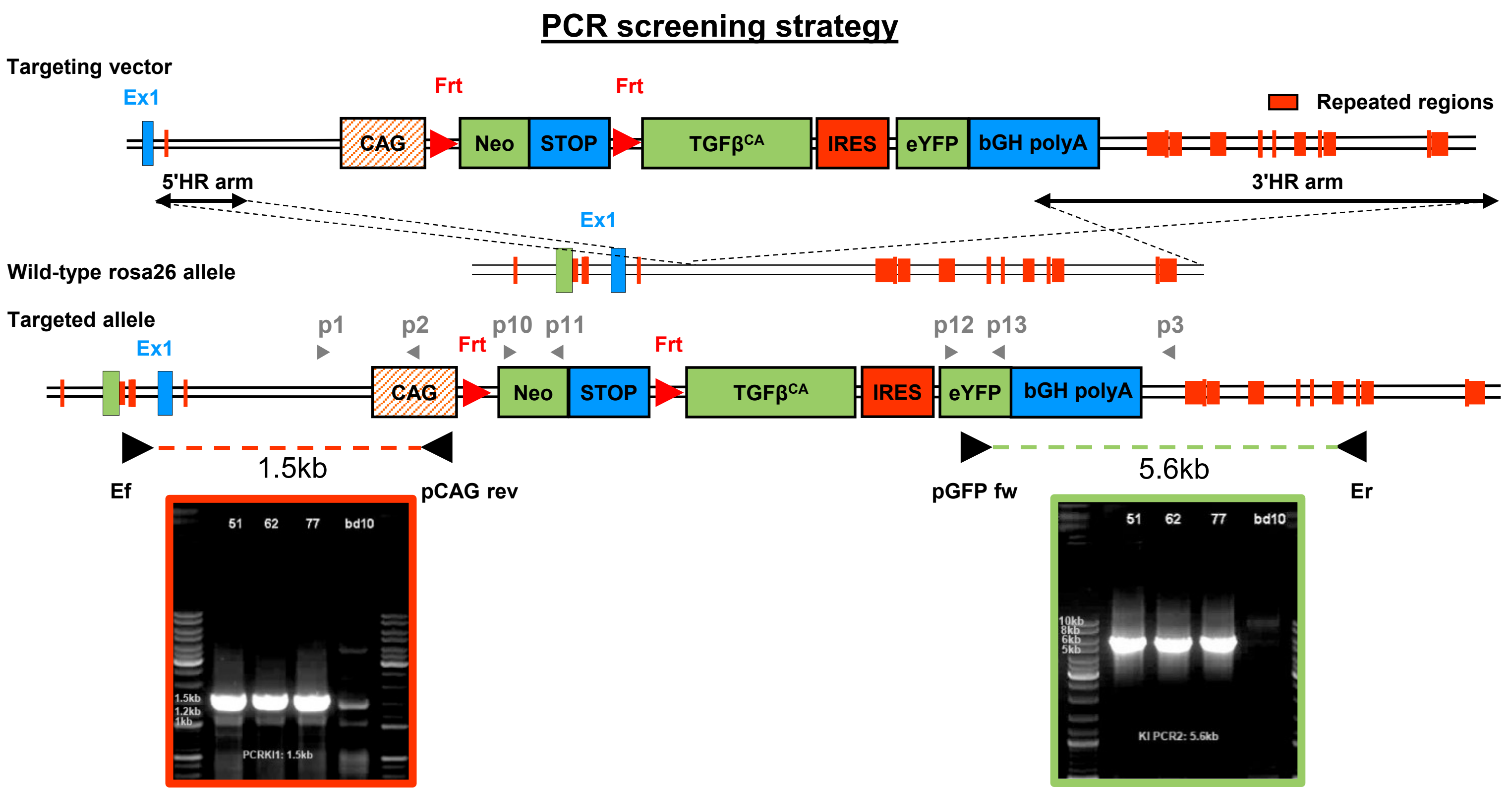
PCR screening strategy
Targeting vector
Ex1
Frt
Frt
CAG
Neo
STOP
TGFβCA
IRES
eYFP
bGH polyA
Repeated regions
5'HR arm
3'HR arm
Ex1
Wild-type rosa26 allele
Targeted allele
p1
p2
p10
p11
p12
p13
p3
Ex1
Frt
Frt
CAG
Neo
STOP
TGFβCA
IRES
eYFP
bGH polyA
1.5kb
5.6kb
Ef
pCAG rev
pGFP fw
Er
51 62 77 bd10
1.5kb
1.2kb
1kb
PCRKI1: 1.5kb
51 62 77 bd10
10kb
8kb
6kb
5kb
KI PCR2: 5.6kb

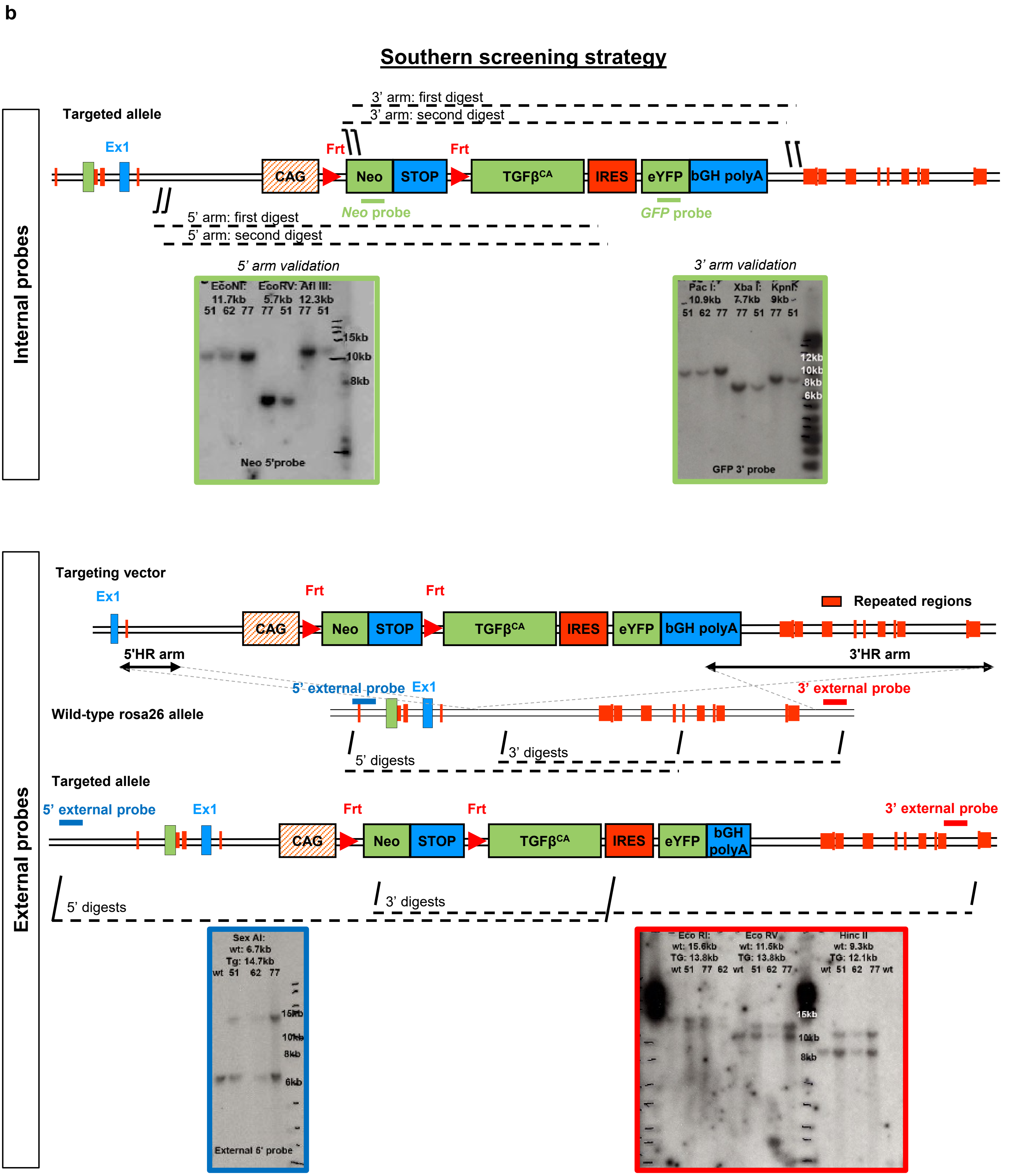

Fig. S3: Validation of *$^{FSF}TGF\beta^{CA}$ ES* clones. a) PCR screening strategy using 2 different set of primers (Ef/pCAG rev and pGFP fw/Er). **b)** Southern blot screening strategy. Top panel, 2 internal probes (Neo and GFP) and 6 different digests are used to validate correct HR event. 3 digests validate the 5' insertion, 3 other digests validate the 3' insertion. Bottom panel, 2 external probes (5' and 3'). 1 digest was used with 5' probe and 3 different digests were used with 3'probe. HR, Homologous Recombination.

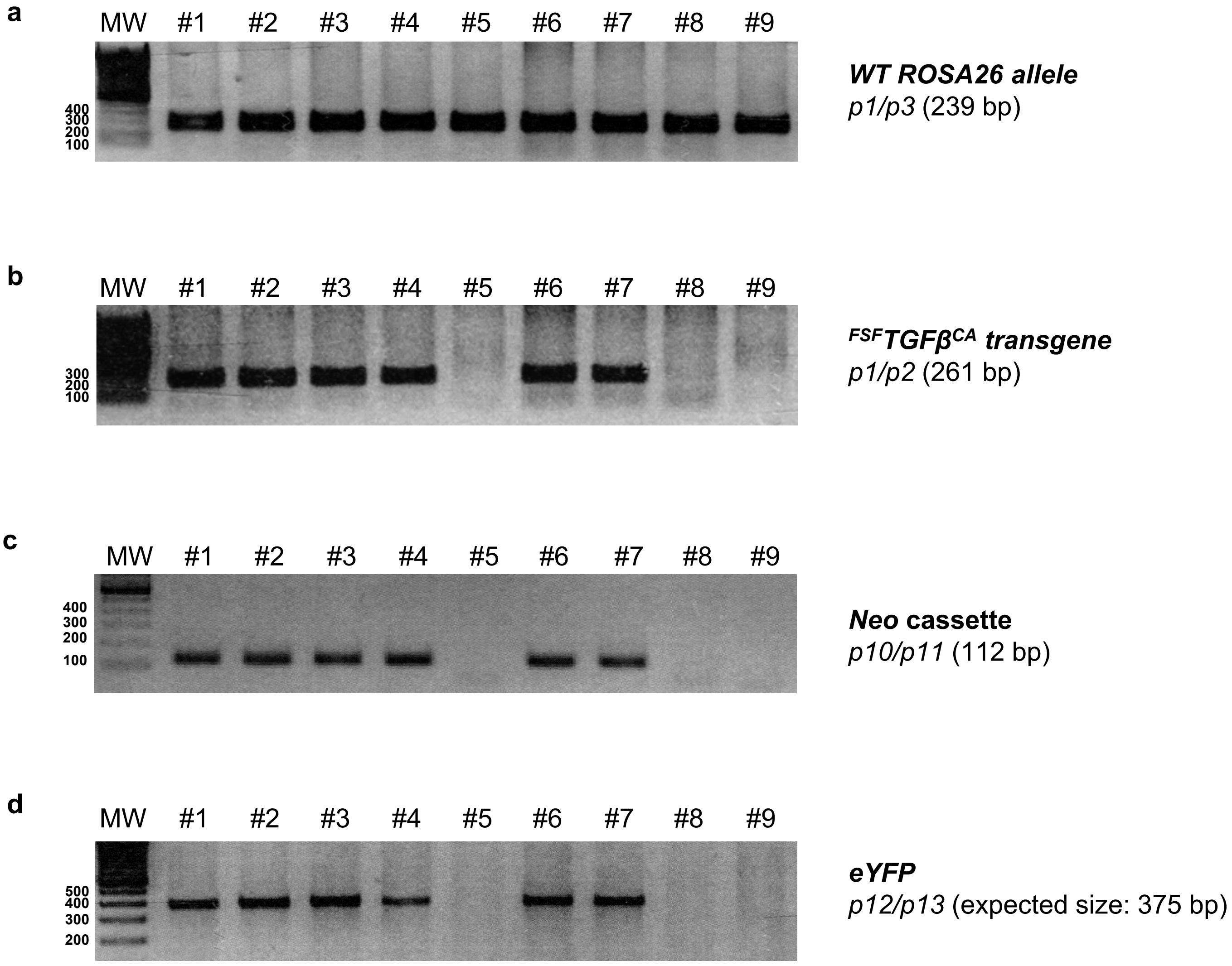

Fig. S4: Genotyping of F1 [^{FSF}TGFβ^{CA}] heterozygous individuals. Primers are positioned on the sequence in Fig. S3a. **a)** *ROSA26* wild-type allele genotyping. **b)** *CAG* promoter genotyping. **c)** *Neo* transgene genotyping. **d)** *eYFP* transgene genotyping. In a, b, c and d, the name of primers are indicated on the right of each gel. Primers sequences are shown in table 1.
